# A multi-task graph-clustering approach for chromosome conformation capture data sets identifies conserved modules of chromosomal interactions

**DOI:** 10.1186/s13059-016-0962-8

**Published:** 2016-05-27

**Authors:** Alireza Fotuhi Siahpirani, Ferhat Ay, Sushmita Roy

**Affiliations:** Department of Computer Sciences, University of Wisconsin, Madison, 53717 WI USA; La Jolla Institute for Allergy and Immunology, 9420 Athena Circle, La Jolla, 92037 CA USA; Wisconsin Institute for Discovery, University of Wisconsin, Madison, 53717 WI USA; Department of Biostatistics and Medical Informatics, University of Wisconsin, Madison, 53717 WI USA

## Abstract

Chromosome conformation capture methods are being increasingly used to study three-dimensional genome architecture in multiple cell types and species. An important challenge is to examine changes in three-dimensional architecture across cell types and species. We present Arboretum-Hi-C, a multi-task spectral clustering method, to identify common and context-specific aspects of genome architecture. Compared to standard clustering, Arboretum-Hi-C produced more biologically consistent patterns of conservation. Most clusters are conserved and enriched for either high- or low-activity genomic signals. Most genomic regions diverge between clusters with similar chromatin state except for a few that are associated with lamina-associated domains and open chromatin.

**Electronic supplementary material:**

The online version of this article (doi:10.1186/s13059-016-0962-8) contains supplementary material, which is available to authorized users.

## Background

The three-dimensional (3D) organization of the genome is emerging as an important layer in the regulation of gene expression [1–10]. Recent advances in high-throughput chromosome conformation capture (3C, particularly 4C, 5C, and Hi-C) technology allow us to examine the 3D organization of a genome in an unbiased and comprehensive manner [1, 8]. Genome-wide 3C data sets are becoming increasingly available for multiple species and tissues and have enabled us to examine the folding and organizational principles of the genome and identify long-range interactions among genomic loci [1, 11]. In particular, studies in yeast have shown that such long-range interactions are enriched for loci involving tRNA genes, centromeres, early origins of replication [4], and transcription factories for regulation of gene expression [12]. In mammalian systems, such interactions are organized into architectural units known as compartments and topologically associated domains (TADs). While the interactions can be cell-type- [13] or species-specific [14, 15], the compartments and TADs are likely conserved across developmental stages [3, 16] and across species [14]. However, our understanding of the extent of conservation and context-specificity of these interactions is incomplete.

The availability of genome-wide 3C data sets for multiple species and tissues gives us the unique opportunity to compare chromatin organization across tissues and organisms to identify the principles of this organization. In parallel, statistical techniques have been developed to normalize these data, identify significant interacting genomic loci [17–19], and identify different types of organizational units from these data [20]. Clustering and dimensionality reduction approaches, in particular, have emerged as important analytical tools for Hi-C data [8, 9, 19, 21]. Rao et al. clustered high-resolution in situ Hi-C data and found six main clusters exhibiting distinct patterns of chromatin state [9]. Principal component analyses of Hi-C data for each chromosome revealed a compartment structure [8], where regions within each compartment are more likely to interact than regions from two different compartments. Imakaev et al. found that the first eigenvector of the genome-wide normalized contact count map exhibited similar properties as the two-compartment model [21]. The second and third eigenvectors exhibited variation along the chromosomal arms, with increased magnitude in the centromeric and telomeric regions for the second and third eigenvectors, respectively.

While current clustering and dimensionality reduction techniques have provided useful insights into genome organization, there are several key issues that need to be addressed. First, unlike traditional functional genomics data such as genome-wide mRNA level or histone modification measurements, 3C data specify contact counts among pairs of genomic loci. A graph-based representation provides a natural representation of these Hi-C data [22] and incorporating Hi-C interaction information as a graph prior was recently shown to improve chromatin-mark-based genome segmentation and annotation [23]. Graph-clustering methods, such as spectral clustering [24, 25], when applied to graph data, are more advantageous than using conventional clustering. However, to our knowledge, graph-clustering methods, especially across multiple cell types and species, have not been explored with Hi-C data. It is currently unknown whether such methods have any advantages over traditional clustering methods that do not capture the graph nature of 3C data.

The second issue is that methods that systematically compare these maps across multiple tissues or multiple organisms are scarce [3]. In particular, given such contact count matrices from two or more cell types, tissues, or organisms, it is not immediately clear how to identify clusters simultaneously in both cell types and also compare them to identify common and context-specific patterns. The systematic comparison of the general 3D organization of the genome across multiple conditions, cell types, and organisms is still a largely unexplored computational challenge.

In this paper, we first perform a comprehensive analysis of different clustering approaches (hierarchical, *k*-means, and spectral) using different distance measures. Our analysis shows that spectral clustering methods tend to outperform existing non-graph-based methods, producing higher quality clusters based on statistical enrichment of multiple one-dimensional regulatory genomic signals. We next develop a multi-task version of our spectral clustering algorithm and apply it to Hi-C data in four cell lines, two each from human and mouse. Compared to an independent clustering method, our multi-task clustering method finds more biologically consistent patterns of conservation and divergence. Using the inferred clusters, we perform a systematic comparative study of the extent of conservation and divergence in chromosome contact preferences between matched cell lines of different species, and between cell lines of the same species. Our results indicate that most regions maintain their chromosome contact preferences between cell lines, and regions that diverge between species and cell lines are enriched for lamina-associated domains (LADs) and architectural proteins.

## Results

### Graph-based clustering of Hi-C data recovers better clusters than non-graph-based clustering

To assess the utility of graph-based clustering for Hi-C data over non-graph-based clustering, we compared three algorithms: (1) hierarchical clustering, (2) *k*-means, and (3) spectral clustering. Hierarchical clustering and *k*-means have been used widely to analyze functional genomics data sets such as gene expression [26] and chromatin marks [27]. The spectral clustering algorithm is a graph-based clustering method that clusters the eigenvectors of the Laplacian operator on a graph [25]. For all three clustering methods, we considered different distance metrics: (1) Euclidean distance, (2) Pearson’s correlation, (3) Spearman’s correlation, (4) contact counts, and (5) log2 of contact counts. In total, we had 15 clustering approaches that differed by clustering algorithm and distance metric.

We applied each clustering method to Hi-C data from the human H1 embryonic stem cell (hESC) line [3], binned into 2755 1-Mbp bins. Each method was applied to obtain *k*=10 clusters (Methods). We evaluated the quality of clusters from each clustering method using five different statistical measures: (1) the Davies–Bouldin index (DBI), (2) the silhouette index (SI), (3) the difference in contact counts between regions in the same cluster and between regions from different clusters (delta contact count), (4) the number of clusters enriched for a regulatory signal (e.g. transcription factor occupancy or histone modification), and (5) analysis of variance (ANOVA) of a regulatory signal. DBI measures the within-cluster scatter and is a number between 0 and 1; the lower the value the better the clustering. SI assesses the boundaries of clustering and ranges between −1 and 1; the lower the value the worse the clustering. The Kolmogorov–Smirnov (KS) test was used to assess whether a particular feature was significantly high in a cluster compared to the genomic background. ANOVA was used to examine how well the clusters explain the variation in a particular regulatory signal. DBI, SI, and the delta contact count served as internal validation metrics of clustering that need only the data being clustered, while the number of enriched clusters and ANOVA served as measures of external validation.

**Fig. 1:**
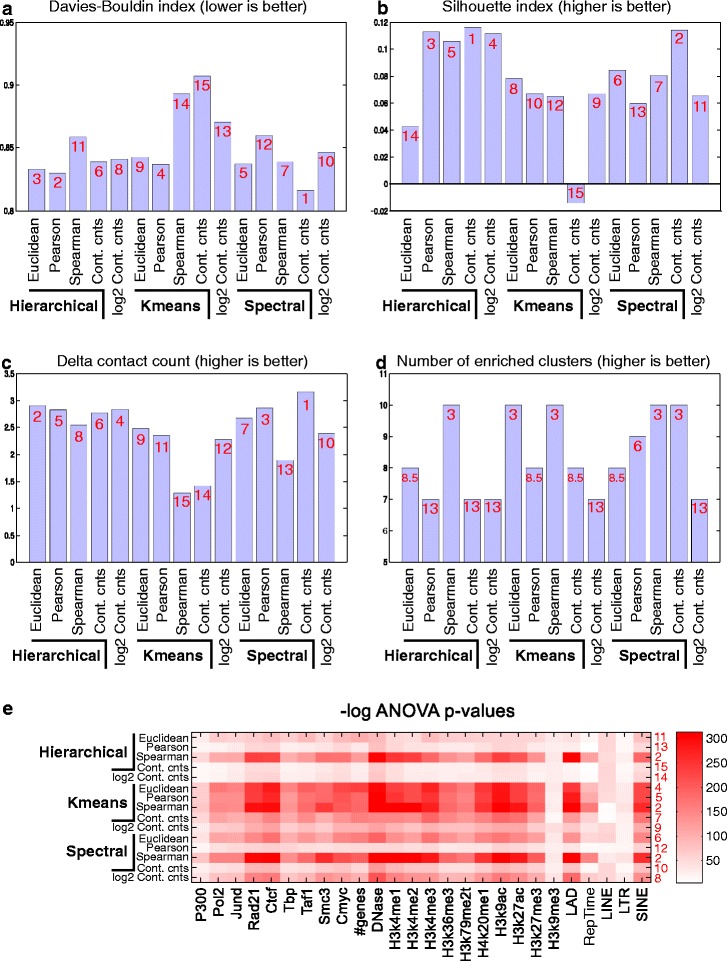
Evaluation of different clustering methods. Shown are a comparison of three clustering methods using different measures: **a** Davies–Bouldin index. **b** Silhouette index. **c** Difference in contact counts within and between clusters. **d** Number of clusters with an enriched regulatory signal. **e** − log*P* value of ANOVA for different regulatory signals. Each clustering method was applied with five different distance/similarity criteria between genomic regions: Euclidean, Pearson’s correlation, Spearman’s correlation, contact count, and log2 of contact count. The numbers (in *red*) correspond to the rank of the methods using the given measure. *Cont. cnts* contact count

**Fig. 2:**
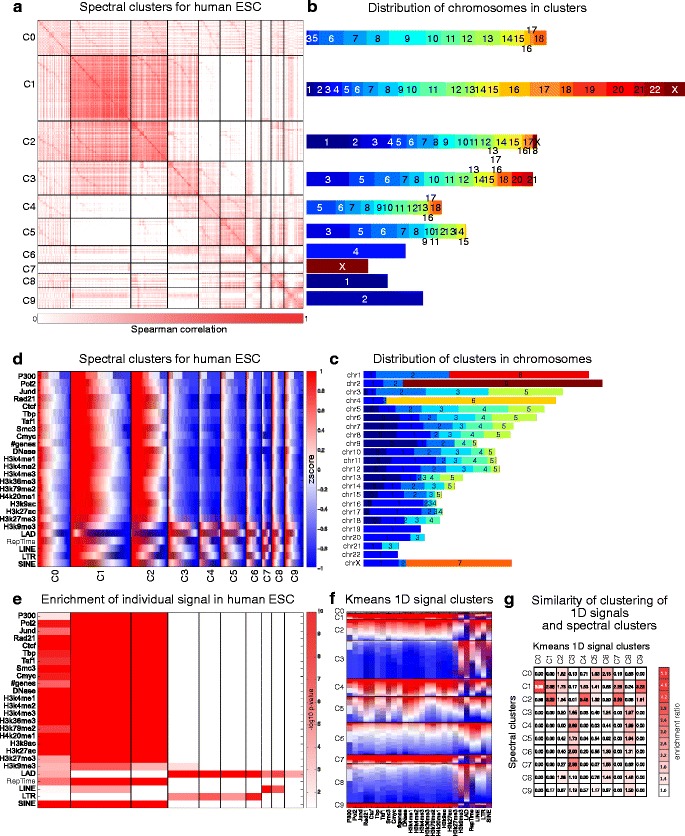
Hi-C clusters for a human embryonic stem cell (ESC) from spectral clustering (Spearman’s >0). **a** Heat map of Spearman’s correlation matrix of contact counts of human ESC, reordered according to spectral cluster assignments. **b** Bar plot showing the distribution of chromosomes in the clusters. The height of the bars corresponds to the size of the clusters. Colors and numbers correspond to different chromosomes. **c** Bar plot showing the distribution of clusters in the chromosomes. The height of the bars corresponds to the size of the chromosomes. Colors and numbers correspond to different clusters. **d** Different regulatory features grouped according to the spectral cluster assignments. We sorted each feature in each column separately to show better the enrichment of the features in the clusters. The features were standardized using *z* score. **e** − log10 of KS test *P* values. For each signal in each cluster, we compared the signal values inside and outside the cluster using the KS test to check if the values inside the cluster are significantly higher than the values outside the cluster. Note that for visualization, clusters were reordered based on their enrichment patterns to put clusters with similar patterns close to each other. **f** The same standardized feature matrix, clustered using *k*-means. **g** Enrichment ratio between spectral clusters and *k*-means clusters. The ratio between cluster *c*_*i*_ of spectral clustering and *c*_*j*_ of *k*-means was defined as (*o*/*N*)/(*K*/*M*) where *M* is the total number of bins, *K* is the number of bins in *c*_*j*_, *N* is the number of bins in *c*_*i*_, and *o* is the number of bins shared between *c*_*i*_ and *c*_*j*_. *1D* one-dimensional, *ESC* embryonic stem cell, *KS* Kolmogorov–Smirnov

A comparison of different clustering approaches showed considerable variation among the different methods (Fig. 1). For example, using DBI and SI, hierarchical clustering with 1-Pearson’s correlation as a distance measure was among the best performing methods (Fig. 1a, b), but it was among the worst when using the number of enriched clusters or ANOVA (Fig. 1d, e). To compare the different clustering approaches across all these measures, we, therefore, ranked each method on a scale of 1 to 15 (appropriately adjusting ties) on each of the evaluation metrics, and computed the average rank for each method. Based on the average rank, the top five methods were spectral clustering on contact count (1), hierarchical clustering with 1-Spearman’s correlation as the distance measure (2), spectral clustering with Spearman’s correlation (3), spectral clustering with Euclidean distance (4), and *k*-means using Euclidean distance (5). Thus, three among the top five ranking methods were spectral clustering variants. We next inspected the patterns of enrichment in the clusters from each method. We found that clusters obtained from spectral clustering with Spearman’s correlation (Fig. 2) were most distinct in their patterns of enrichment compared to the other variants of spectral clustering (Additional file 1: Figure S1) and hierarchical clustering (Additional file 1: Figure S2, Additional file 2). In particular, spectral clustering with Spearman’s correlation found three clusters that were significantly enriched with open chromatin signatures (described in detail in the next section, Fig. 2d). In contrast, clusters from hierarchical clustering were unbalanced and all the activating marks were concentrated in one cluster. Thus, the clusters obtained from spectral clustering on Spearman’s correlation are likely more biologically meaningful based on external validation measures and are comparable to hierarchical clustering approaches for internal validation metrics. Based on these observations, we selected spectral clustering (Spearman’s correlation) for our subsequent analysis. We note that our clustering framework is flexible and can use other definitions of graph weights as well.

### Spectral clustering can incorporate both *cis* and *trans* interactions and identifies two major types of clusters

Inspection of the chromosomal coverage of our clusters in hESC showed that most (six of ten) clusters cover multiple chromosomes, revealing *cis* and *trans* interactions (Fig. 2a, Methods). Spectral clustering of only *trans* interactions finds a similar number of multi-chromosomal clusters suggesting that our clustering is robust and that intra-chromosomal interactions do not overshadow the inter-chromosomal interactions (Additional file 1: Figure S3, Additional file 2).

To interpret our clusters functionally and relate them to downstream gene expression programs, we tested our clusters for statistical enrichment of multiple genome-wide regulatory signals including chromatin marks (H3K4me1, H3K4me2, H3K4me3, H3K36me3, H3K79me2, H4K20me1, H3K9ac, H3K27ac, H3K27me3, and H3K9me3), LADs, early versus late replication timing (RepTime), general transcription factors (POLII, TAF, TBP, CTCF, P300, and CMYC), cohesin components (RAD21 and SMC3), open chromatin from DNase I hypersensitivity assays, number of genes, and various classes of repeat elements [short interspersed nuclear elements (SINEs), long interspersed elements (LINEs), and long terminal repeats (LTRs)].

We found that clusters C0, C1, and C2 were significantly enriched with gene-rich regions, open chromatin (DNase I), SINE, and activating and repressive marks, with the exception of H3K9me3, which varied between the clusters (KS test *P*<0.05, Fig. 2d, e). Cluster C0 was also moderately enriched for LADs while C1 and C2 were depleted in LADs. The remaining seven clusters were associated with LADs, LINEs, and LTRs, and were depleted for genes and chromatin marks. The clusters comprising entirely regions from one chromosome were associated with LADs and either LINE (C7 and C8) or LTRs (C6). We also observed that SINE and LINE enrichments are exclusive: SINEs tend to be with clusters with high genomic activity (i.e. enriched for different chromatin marks and gene-rich regions), while LINE and LTR elements are associated with LAD clusters. Our observation that the clusters associated with gene-rich regions are depleted in LADs and clusters associated with gene-poor regions are enriched for LADs is in agreement with previous studies that showed LADs are relatively gene poor [28]. Because the clusters appeared to be discriminated based on activity, we asked if DNase I footprints can explain the association of all other marks. We observe significant conditional mutual information between each signal and the clustering assignments given DNase I, which suggests there is information to be gained by the clustering that is not captured in the DNase I signal (Additional file 1: Methods, Additional file 1: Figure S4). Furthermore, the observed values of the different evaluation metrics (SI, DBI, and delta contact counts) are significantly higher than random, suggesting that we are not over-clustering (Additional file 1: Methods and Additional file 1: Figure S5).

In parallel, we clustered the genomic regions using *k*-means on their one-dimensional signal profiles (Fig. 2f) and compared these clusters to the spectral clusters based on a hypergeometric test. Several of the Hi-C clusters were mutually enriched in these *k*-means clusters (Fig. 2g), suggesting that these two partitions of the data are mutually consistent with each other. For example, the spectral clusters C1 and C2 (with high genomic activity) had significant overlap with the *k*-means clusters C0 and C4. However, the Hi-C clusters do not have a one-to-one mapping with the one-dimensional signal *k*-means clusters (e.g. the C3 *k*-means cluster had significant overlap with the C4, C6, and C7 spectral clusters), suggesting that the Hi-C clusters capture additional information that is specific to the 3D organization of the genome. We repeated this analysis for Hi-C data in a mouse ESC (mESC) line from Dixon et al. [3] (Additional file 1: Figure S6 and Additional file 2), and observed similar patterns, suggesting that our clusters are capturing generalizable properties of chromosomal organization.

To test the sensitivity of our conclusions to fixed-sized bins, we also considered regions defined by TADs. Briefly, we aggregated the counts in TADs defined in Dixon et al. [3] and clustered the resulting matrix (Additional file 1: Methods). We observed similar patterns of enrichment in these clusters and found that 43 % of the total bases were co-clustered when using a fixed bin size and clusters of TADs (Additional file 1: Figure S7, Additional file 2). We also repeated our analysis of the hESC data for multiple resolutions, 100 and 500 kbp. There was a significant overlap of base pair coverage between clusters at different resolutions (64 % for 100 and 500 kbp, 53 % for 100 kbp and 1 Mbp, and 71 % for 500 kbp and 1 Mbp), which is significantly greater than random (Additional file 1: Table S1). Furthermore, we could find a one-to-one mapping for the majority of the clusters, and the mapped clusters also exhibited similar patterns of enrichment as the 1-Mbp regions (Additional file 1: Figure S8).

### Hi-C data clusters from spectral clustering recapitulate known and novel higher-order organizational units

To examine the relationship between our spectral clusters and major chromosomal architectural units such as compartments on individual chromosomes [8], we applied *k*=2 clustering to our data. A compartment is defined by a subset of regions on a chromosome that densely interact with each other, but are depleted for interactions with other regions on the chromosome. We obtained the cluster assignment for all regions in a chromosome and compared these cluster assignments to the compartments (Additional file 1: Figure S9 and Methods). The majority of the chromosomes (except for chromosomes 16, 19, 20, 21, and 22) were partitioned into two clusters by our approach, indicating the presence of compartment-like structures in our clustering results. Pairs of regions that were clustered together by spectral clustering tended to be in the same compartment as assessed by two independent measures of co-clustering. In the majority of the chromosomes (18 out of 23), these measures are significantly higher than what is expected by chance (*F* score: 60–80 %, *t* test *P*<3.49×10^−5^, and Rand index: 50–80 %, *t* test *P*<1.45×10^−5^, Additional file 1: Figure S9), suggesting that spectral clustering with *k*=2 can also recover aspects of compartments. Chromosomes 16, 19, 20, 21, and 22 are not detectable as separate clusters with *k*=2, likely because they tend to co-localize in the nucleus [8]. The application of the spectral clustering method at higher resolution (e.g. 40 kbp instead of 1 Mbp), can recover TAD-like structures (Additional file 1: Figure S10a, b, c, d, and Additional file 1: Methods). In addition, applying the clustering method to each chromosome separately can also recover clusters with significant overlap with the compartment (Additional file 1: Figure S10e, f, g). These results further suggest that graph-based clustering approaches can be a general and powerful approach for recovering different organizational units of the genome, spanning both *cis* (within one chromosome) and *trans* (between chromosome) interactions.

### Arboretum-Hi-C: A multi-task spectral clustering algorithm for comparative analysis of Hi-C data

**Fig. 3:**
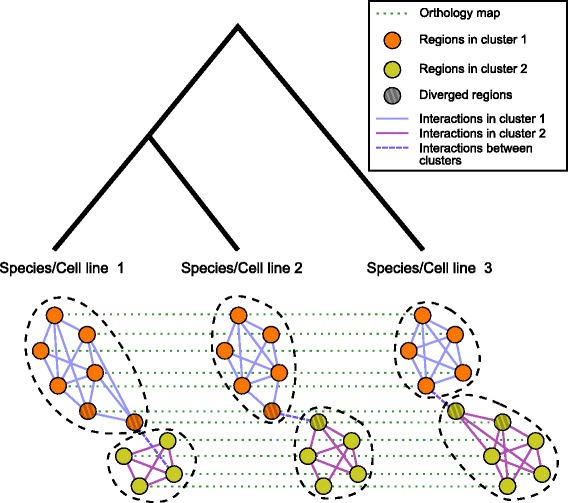
Overview of Arboretum-Hi-C. Given *n* graphs of genomic regions, each representing a Hi-C data set in *n* species or cell lines, our method clusters the regions based on their interactions, while exploiting the relatedness (shown as the hierarchy) among the data sets. The figure shows an example of three Hi-C data sets with two clusters. The example shows that some regions keep their cluster assignments in all species, while other regions (diverged regions) change their cluster based on changes in their interactions with other regions. *Dashed lines* represent a mapping of regions from one data set to another. For cell lines of the same species, this mapping is trivial as the same regions are studied. For multiple species, this mapping requires one to find orthologous regions between species

Having determined that spectral clustering is a powerful approach for analyzing Hi-C data from one cell line, we next developed a new approach, Arboretum-Hi-C, to compare systematically the 3D organization across multiple cell types and species. Arboretum-Hi-C combines two clustering strategies: spectral clustering and multi-task clustering (Fig. 3). Multi-task clustering is a special case of multi-task learning [29], where the goal is to solve multiple learning tasks simultaneously. Arboretum-Hi-C takes as input *n* different Hi-C data sets (*n*=3 in Fig. 3), representing possibly different cell lines or species, a tree describing the hierarchical relationship between the data sets, the number of clusters *k*, and a mapping of regions between the different data sets. The Hi-C data sets represent observed data as the leaves of the tree (Fig. 3). As output, Arboretum-Hi-C returns the cluster assignments of regions in each Hi-C data set. Arboretum-Hi-C is based on a previous multi-task clustering approach, Arboretum [30], which uses a generative probabilistic model to cluster expression data from multiple species while accounting for the hierarchical relationships among the species as described by a phylogenetic tree (Methods). However, instead of expression matrices at each leaf node, we now have Hi-C interaction graphs. Edges in these graphs are weighted, with edge weights corresponding to Spearman’s correlation since this gave the best results among different distance metrics. However, our general approach is applicable to different definitions of edge weight (e.g. contact count between a pair of regions). To cluster these graphs, we apply Gaussian mixture model-based clustering to the first *k* eigenvectors of each graph’s Laplacian (Additional file 1: Methods).

### Major modules of chromosome contact interactions are shared between human and mouse cell lines

We applied Arboretum-Hi-C to two human and two mouse cell lines that were studied in Dixon et al. [3]. Two of these cell lines represent the undifferentiated ESC state in both organisms (hESC and mESC, respectively), and the other two cell lines represent examples of a terminally differentiated cell state (IMR90 human fibroblasts and mouse cortex, referred to as hIMR90 and mCortex, respectively). We first examined 1318 1-Mbp human and mouse regions that constitute one-to-one orthologous regions (Methods). Results at a higher resolution (500 kbp) are described subsequently.

We considered two possible hierarchical relationships of these four data sets (Additional file 1: Figure S11 and Additional file 1: Methods) and used the probabilistic framework of Arboretum-Hi-C to select between these two trees. In one tree, the cell lines from the same species were closer to each other, and in the other, the embryonic cell lines from the two species were closer to each and the differentiated cell lines were closer to each other. We observed that the first tree, in which the Hi-C data within a species were closer to each other, had a greater data likelihood (Additional file 1: Figure S11). Therefore, we performed our subsequent analysis with this tree topology.

**Fig. 4:**
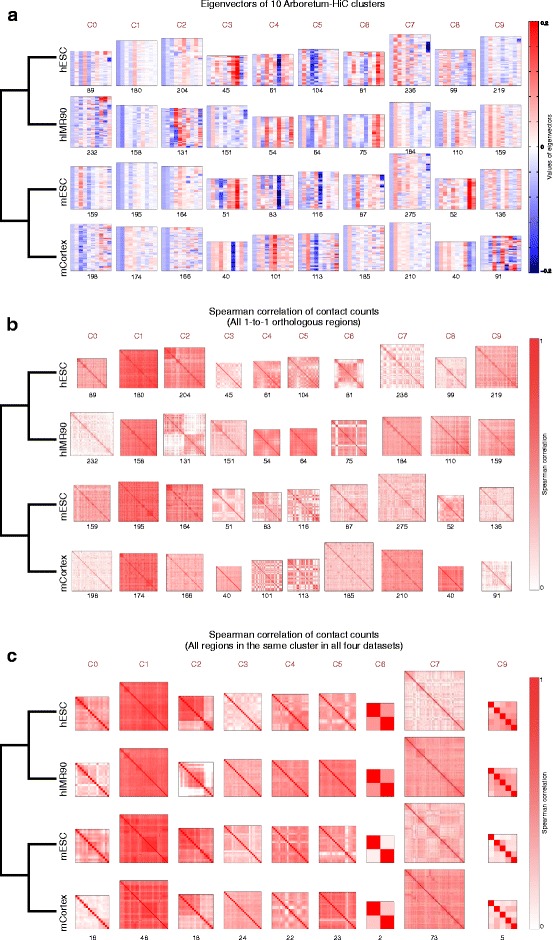
Results of Arboretum-Hi-C on four Hi-C data sets for two human cell lines, hESC, mESC, hIMR90, and mCortex. **a** Shown are the eigenvectors of the Laplacian of each Hi-C-derived graph in each of the ten clusters (major columns). The *red numbers* above the heat maps denote the size of the clusters. **b** Spearman’s correlation heat maps of the ten clusters. The order of the rows is the same as in **(a)**. The numbers correspond to the size of the clusters. **c** Same as **(b)**, but restricted to the bins with the same cluster assignment in all cell lines. Cluster C8 did not have any regions that were common in all four data sets. *hESC* human embryonic stem cell, *hIMR90* IMR90 human fibroblast, *mCortex* mouse cortex, *mESC* mouse embryonic stem cell

Application of Arboretum-Hi-C to these four data sets identified ten clusters of interacting regions, several of which exhibited conserved patterns of interactions (Fig. 4). The multi-task clustering framework of Arboretum-Hi-C provides a correspondence between clusters of one cell line/species to the clusters of another cell line/species. That is, cluster C*i* from hESC would correspond to cluster C*i* of mESC (and all other data sets examined), where *i* ranges from 0 to *k*−1. This correspondence or mapping of clusters between different data sets (as further described and validated below) enables a systematic comparison of patterns of interactions and the regions that participate in these interactions. We visually examined the patterns of these clusters based on the eigenvectors (Fig. 4a) as well as Spearman’s correlation matrices for regions in each cluster (Fig. 4b). Several clusters exhibited conserved patterns of eigenvectors and interactions across all four data sets (C1 and C2), while some clusters were more similar between cell lines of the same species [C6 (human) and C5], and some clusters captured similarity in the ESC state between species (C3 and C4).

**Fig. 5:**
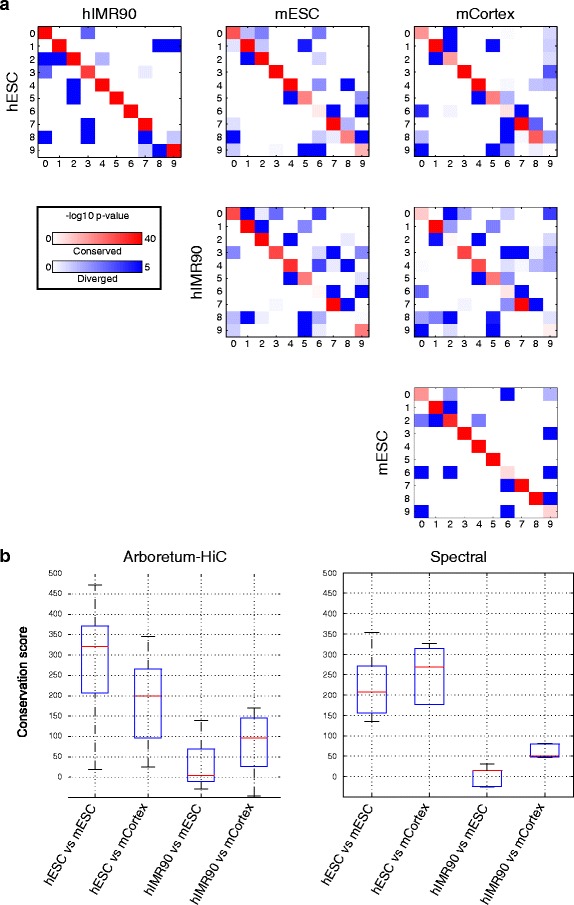
**a** Overlap between clusters from four cell lines inferred by Arboretum-Hi-C. Each *red*-*blue* matrix shows the extent of similarity between clusters from pairs of cell lines or species as measured by − log10*P* value of a hypergeometric test. *Diagonal elements* represent clusters of the same ID and are shown in *red*. *Off-diagonal elements* are shown in *blue*. The intensity of *red* and *blue* is proportional to the extent of similarity between pairs of clusters. **b** The distribution of conservation score between pairs of cell lines estimated from multiple random initializations of Arboretum-Hi-C and independent spectral clustering. The conservation score for Arboretum-Hi-C was defined as the sum of diagonal elements minus off-diagonal elements of the matrices from **(a)**. Because independent spectral clustering does not give a mapping of cluster assignments across data sets, we first matched cluster IDs based on maximal overlap of regions using the Hungarian algorithm (Methods). *hESC* human embryonic stem cell, *hIMR90* IMR90 human fibroblast, *mCortex* mouse cortex, *mESC* mouse embryonic stem cell

To examine the extent of conservation at the region level, we examined these clusters in two ways. First, we extracted the core conserved set of regions by obtaining those regions that were in the same cluster in all species and cell lines (Fig. 4c). We observe a striking pattern of conservation of interactions in this conserved set of regions. For some clusters, this represented a high fraction of their elements (>30 % for clusters C3, C4, and C7), or a moderate fraction (10–30 % for clusters C0, C1, C2, and C5), while for some clusters this represented a small fraction (<10 % for clusters C6, C8, and C9). Cluster C3 was the most conserved, with 44 % of its regions in the conserved core set. Second, we compared the clusters, one pair of cell line/species at a time, using the significance of overlap of orthologous regions of one cluster from one species (or cell line), and another species (or cell line). We quantified the overlap in orthologous regions using the negative log of the hypergeometric test *P* value as described in Roy et al. [30], and visualized them using red-blue heat maps (Fig. 5a), for every pair of species or cell lines. The off-diagonal elements of the heat map denote the shared chromosomal organization between clusters of different IDs, and the diagonal elements measure the extent of conservation between clusters of the same ID (Fig. 5a, red-blue heat maps). We found that between hESC and mESC (same cell type but different species), there were a larger number of strong red diagonal elements compared to hESC and mCortex.

To compare the extent of conservation between the clusters identified by Arboretum-Hi-C to clusters identified by applying spectral clustering to the data sets independently, we calculated the difference in the diagonal elements and off-diagonal elements for every pair of Hi-C data sets over multiple random initializations of the algorithm. We find that using Arboretum-Hi-C there is greater conservation between clusters (Fig. 5b box plot of Arboretum-Hi-C clusters) of the matched cell lines (hESC vs mESC) than between different cell lines (hESC vs mCortex). In contrast, independent clustering of the Hi-C data using non-multi-task spectral clustering did not discriminate between the cell lines and estimated a similar extent of conservation for both matched and different cell lines (Fig. 5b). Overall, the patterns of conservation and divergence from the non-multi-task clustering may not be as biologically meaningful as those from Arboretum-Hi-C.

**Fig. 6:**
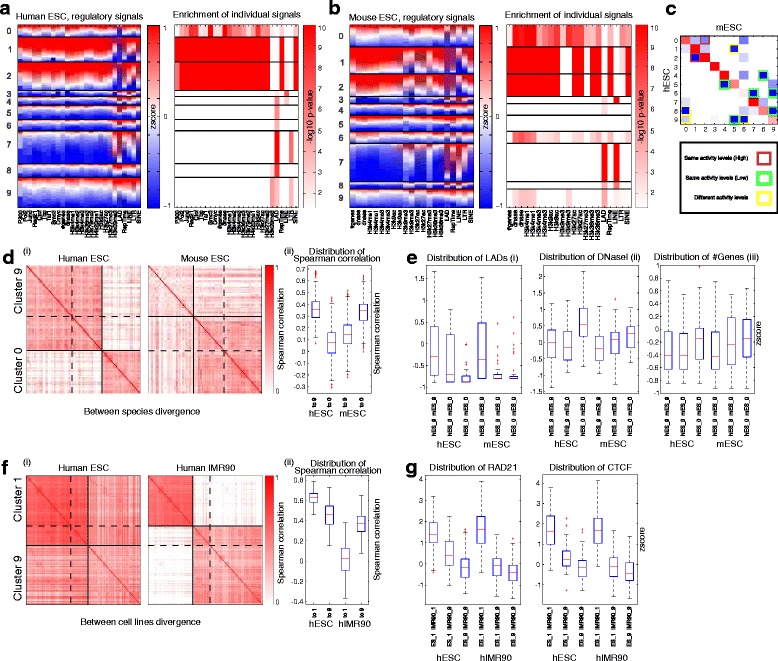
Conservation and divergence of Arboretum-Hi-C clusters of human and mouse ESCs. **a** On the *left* is a heat map of different regulatory features enriched in human ESC, reordered according to the Arboretum cluster assignments, and on the *right* is the heat map of − log10 of KS test *P* values (like Fig. 2). **b** The same as (**a**) for mouse ESC. **c** Heat map of overlap between clusters of human and mouse ESCs. The *off-diagonal elements* are highlighted based on the type of divergence (between clusters of the same activity level or between clusters of different activity levels). **d** Example of module divergence between C9 and C0 in human and mouse ESCs. (**i**) Heat map of Spearman’s correlation of contact counts for the regions that are in C9 or C0 in human and mouse ESC lines. *Solid lines* show the demarcation of regions that are in the same cluster in both human and mouse, while *dashed lines* show regions that have switched clusters between C9 and C0 in human and mouse. (**ii**) Box plots of distribution of correlation from 29 diverged regions to the regions that stay in C9 and C0 in both human and mouse. **e** Distribution of (**i**) LADs, (**ii**) DNase I peaks, and (**iii**) number of genes in regions that are conserved or switch between clusters. (**i**) The first three box plots for LADs show human regions that remain C9 in both human and mouse (*hESC_9 mESC_9*), regions that switch from C9 to C0 (*hESC_9 mESC_0*), and the regions that are in C0 in both human and mouse (*hESC_0 mESC_0*). The remaining three box plots shows the LAD distribution in mouse ESC for regions that remain in C9 in both human and mouse, the regions that have switched to C0, and finally, those that remain in C0 in both. (**ii**) and (**iii**) are the same as (**i**), showing DNase I and number of genes. **f** Like **d** for the regions that diverge between C1 and C9 in human ESC and IMR90 cells. **g** Like **e**, showing the distribution of RAD21 and CTCF peaks in regions exhibiting the same and difference cluster assignments in hESC and hIMR90. *ESC* embryonic stem cell, *hESC* human embryonic stem cell, *hIMR90* IMR90 human fibroblast, *LAD* lamina-associated domain, *mESC* mouse embryonic stem cell

To assess the extent to which conserved chromosomal modules exhibit similar regulatory signals and validate the mapping of clusters between data sets identified by Arboretum-Hi-C, we examined these clusters for enrichment of regulatory signals (Fig. 6). Arboretum-Hi-C mESC and hESC clusters of the same ID exhibited similar patterns of enrichment. In particular, clusters C0, C1, and C2, in both hESC and mESC were associated with gene-rich, open chromatin, chromatin mark modified, LAD-depleted regions (Fig. 6a, b). Similarly, clusters C3, C4, C7, C8, and C9 were gene poor and associated with LADs and repeat elements. SINEs tend to be associated with gene-rich, active chromatin, mark modified regions, while LINEs and LTRs are associated with LADs and gene-poor regions. Overall, we found that Arboretum-Hi-C clusters in both species could be grouped into clusters with high (C0, C1, and C2) and low genomic activity (C3, C4, C7, C8, and C9). While some clusters exhibited additional signal enrichment (e.g. mESC C9, H3K9me3, and DNase I), clusters with the same ID exhibited similar patterns of enrichment, despite not being completely orthologous, thus validating the correspondence of chromosomal cluster IDs of Arboretum-Hi-C.

To assess the effect of bin size in the definition of orthology mapping of the regions and the subsequent Arboretum-Hi-C analysis, we repeated our experiments at a higher resolution of 500 kbp, clustering a total of 2342 regions. As observed in the 1-Mbp case, we found conserved modules between human and mouse cell lines that could be matched based on their enrichment patterns (Additional file 1: Figure S12a, b and Additional file 2). Furthermore, we observed significant overlap between clusters obtained at 500-kbp resolution and 1-Mbp resolution (Additional file 1: Figure S12c), suggesting that changes in the bin size at this resolution (1 Mbp to 500 kbp) does not significantly affect the resulting clusters. To test whether intra-chromosomal interactions create a bias by overshadowing the inter-chromosomal interactions, we repeated our analysis after removing any interactions that are between regions of the same chromosome in human or mouse (Additional file 1: Methods). As in independent clustering, we observe significant overlap between clusters derived from inter-chromosomal interactions and clusters using both inter- and intra-chromosomal interactions (Additional file 1: Figure S13).

### Changes in chromosome contact modules between human and mouse cell lines

We next examined module divergence between species and module dissimilarity between cell lines by inspecting the off-diagonal elements of the red-blue heat maps in Fig. 5a. This analysis relied on our characterization of clusters into high and low activity described in the previous section. We found that most of the module transitions were between clusters of the same type, that is, from low activity to low activity, or from high activity to high activity (Table 1). However, there were a few examples of transitions between modules with high and low genomic activity that we discuss below.

**Table 1:** Number of significant divergence events between pairs of clusters of different types in each pair of cell lines

		hIMR90	
		High activity	Low activity
hESC	High activity	3	1
	Low activity	4	3
		mESC	
		High activity	Low activity
hESC	High activity	1	1
	Low activity	2	7
		mCortex	
		High activity	Low activity
hESC	High activity	3	1
	Low activity	2	5
		mESC	
		High activity	Low activity
hIMR90	High activity	3	2
	Low activity	0	7
		mCortex	
		High activity	Low activity
hIMR90	High activity	3	2
	Low activity	1	6
		mCortex	
		High activity	Low activity
mESC	High activity	2	1
	Low activity	3	5

*hESC* human embryonic stem cell, *hIMR90* IMR90 human fibroblast, *mCortex* mouse cortex, *mESC* mouse embryonic stem cell

#### Changes in high and low activity modules between species are associated with LADs and chromatin activity

We found three examples of transitions between species-specific modules of different activities. One transition was between hESC C9 and mESC C0 involving 29 regions spanning a total 29 Mbp. The C9 cluster is associated with LADs, whereas C0 is associated with open chromatin and histone modification marks (Fig. 6a, b, c). Comparison of regulatory features of these 29 regions (*hES_9 mES_0*) against regions that maintained their cluster assignment in C9 (*hES_9 mES_9*) and C0 (*hES_0 mES_0*) in hESC showed that these switched regions were less LAD-rich than the regions in cluster C9 (KS test *P*<8.02×10^−2^, Fig. 6e). In mESC, where these regions were assigned to C0, a cluster with high activity, they tended to have a lower propensity of LADs than other regions associated with C9 (KS test *P*<8.66×10^−5^), and more like other elements of C0. These regions exhibit a similar tendency for the number of genes and DNase I elements (Fig. 6e(ii), (iii)). A second transition, also between a high- and low-activity module, was from hESC C8 (low activity) to mESC C0 (high activity, Fig. 6a, b, c) and included 21 regions. In both hESC and mESC, these regions have significantly lower LAD content than the regions with conserved assignments to cluster C8 (KS test *P*<4×10^−3^, Additional file 1: Figure S14a). In addition, in hESCs, these regions have a significantly higher LAD content than regions that are in cluster C0 in both species (KS test *P*<1×10^−4^). Similarly, DNase I and gene count in human regions that switch are intermediate between the conserved members of C8 and C0 in both human and mouse (Additional file 1: Figure S14b, c). The third transition was in a different direction involving regions in a high-activity module in human (C1) and a module C5 in mouse, which was not significantly enriched for any signals in mouse, but is likely a low-activity cluster based on the enrichment profile of the orthologous human C5. Although, the human regions that transitioned to module C5 in mouse did not exhibit a significantly different distribution in LADs, they exhibited a significantly depleted pattern of enrichment for DNase I (KS test *P*<7.89×10^−3^) and gene count (KS test *P*<2.18×10^−2^ when comparing diverged regions to C1 in mouse, Additional file 1: Figure S14d,e). Overall, these results suggest that the regions that switch their chromatin interaction preference between species are associated with different one-dimensional signals than the regions that maintain their interaction preference between species.

#### Changes in module assignment between cell lines are associated with CTCF and RAD21 binding sites

In addition to transitions in modules between species, we found several examples of transitions between clusters with high and low activity among cell lines of the same species (five within human and four within mouse, Table 1). One example of such transitions is between cluster C9 (low activity) of hIMR90 and cluster C1 (high activity) of hESC. Figure 6f shows the pattern of correlation of contact counts for the regions in clusters C1 and C9 and regions that change their cluster assignment. To relate these transitions to the binding profiles of general transcription factors, we examined the distribution of binding of transcription factors measured in both cell lines, namely CEBPB, CTCF, MAFK, POLR2A, and RAD21 (Fig. 6g and Additional file 1: Figure S15). Among these transcription factors, CTCF and RAD21 appeared to discriminate hESC regions that remained in C1 and those that were in C9 in hIMR90 (KS test *P*<6.04×10^−4^ and *P*<1.12×10^−4^, respectively). Similarly, in hIMR90, these regions were more enriched than the regions that were in cluster C9 in both cell lines (KS test *P*<6.20×10^−2^ for CTCF and *P*<3.05×10^−2^ for RAD21). This differential enrichment suggests that CTCF and RAD21, which are known to be major players in chromosomal architecture and organization [31], likely contribute to cell-type-specific behavior between a differentiated and undifferentiated cellular state.

## Conclusions

Chromosome conformation capture (3C) assays [2], such as 4C [32], 5C [33], and Hi-C [8], as well as factor-specific ChIA-PET studies [34], are being increasingly applied to more cell types and species [3, 7, 14, 35–39]. Computational approaches for analyzing such data sets, and more importantly, comparing such maps across multiple tissues, are still in their infancy [20]. Here we performed a systematic analysis of graph-based and non-graph-based clustering methods for Hi-C data. Our comparisons showed that graph-based clustering with different distance metrics tends to outperform non-graph-based clustering, suggesting that incorporating the graph-based nature of Hi-C (and other 3C) data is advantageous for clustering. We developed Arboretum-Hi-C, a novel graph-based multi-task clustering approach to find common and cell-line- and species-specific patterns of interacting chromosomal regions. The multi-task nature of our analysis framework enables us to uniformly map and compare clusters across multiple Hi-C data sets. Furthermore, representing the relationship of these data sets as a tree enables us to study the extent of similarity of the corresponding species or cell lines. Simultaneous clustering of multiple data sets using the Arboretum-Hi-C framework showed that chromosome conformations in mESCs and hESCs are more similar to each other than between human and mouse differentiated cell states.

The ability to match clusters from one cell line or species to another becomes increasingly complicated as the number of cell lines or species increases. Arboretum-Hi-C addresses this challenge by using a multi-task clustering framework that also exploits the hierarchical relationships among cell types and species and where cluster IDs are tied to the topmost node in the hierarchy. Our approach provides a one-to-one mapping between clusters identified across multiple species or cell lines, which enables a systematic comparison of sets of regions across species and cell lines. We validated this one-to-one mapping in mouse and hESC lines by showing that the clusters of the same IDs are also enriched for similar regulatory signals (e.g. C1 of both hESCs and mESCs are associated with gene-poor LAD regions). We observed striking conservation between the modules inferred across the species for matched cell lines, which is consistent with a recent comparative study done in liver for four mammalian species [14]. We note that Arboretum-Hi-C is a data-driven approach and we used the data likelihood to decide between alternative tree topologies that could relate the Hi-C data sets studied.

Our clustering approach also enabled us to study the context specificity of chromosomal interactions within and between species in a single unified framework. A change in cluster assignment between cell lines or between species suggests that those regions interact with other chromosomal regions. Such transitions are likely associated with the overall cell-line-specific or species-specific behaviors. We found that most of these changes are between modules with similar regulatory signals (that is, most transitions are between clusters with low activity and low activity, or high activity and high activity).

The occurrence of CTCF and RAD21 in regions that switch their chromosomal interaction cluster between cell lines is consistent with the role of these proteins as key determinants of the 3D organization of the genome. In particular, CTCF was shown to be associated with the divergence of TADs between species [14]. CTCF is also associated with cell-line-specific changes in TADs [3, 23]. The presence of a LAD in regions that diverged their chromosomal contact preferences suggests a possible role of LADs in contributing raw material to the evolution of regulatory regions. However, with only two species, it is difficult to establish whether the changes in one-dimensional signals are the cause or consequence of the topological reorganization. As the number of species with available Hi-C data increases, we will be able to address these questions in a more principled manner. By comparing differentiated cells and undifferentiated cells from human and mouse, we were also able to examine the extent of conservation between matched cell types. We found that the modules identified in mESCs were more similar to hESCs. While this served as a useful validation of our data-driven clustering, having matched differentiated cell lines would greatly improve the comparative power of our approach.

We demonstrated our approach on relatively large regions (1 Mbp) as well as variable-sized regions defined by TADs (Additional file 1: Figure S7) to enable the identification of large-scale chromosomal interactions that include both *cis* and *trans* interactions. However, our approach can also be applied in *cis* one chromosome at a time to identify TAD-like structures (Additional file 1: Methods and Additional file 1: Figure S10) as well as to find compartments (Additional file 1: Figure S9). These results suggest that this is a powerful and flexible clustering algorithm to identify known and novel chromosomal organizational units. Most of our analysis was done at a relatively coarse resolution, which remains fixed during the clustering procedure. An important extension is to have a flexible multi-resolution clustering algorithm that can adaptively select the distance measure depending upon the resolution.

In summary, we have performed a systematic analysis of different clustering methods for high-throughput 3C data sets that measure the 3D proximity of pairs of genomic regions. We also presented an algorithm to perform clustering across multiple species and identified patterns of significant conservation as well as species-specific and cell-line-specific divergence. As such Hi-C maps become available for diverse cell types and species [9, 14, 40–42], methods such as ours will be increasingly useful for systematic comparisons to identify common and context-specific properties of genome architecture, revealing principles governing the organization of chromatin and its impact on complex phenotypes.

## Methods

### Data set description and pre-processing

We used the publicly available Hi-C data for two human cell lines (H1ES and IMR90) and two mouse cell lines (J1 ES and cortex) from Dixon et al. (GEO accession code GSE35156 [3]). Paired reads were aligned to the reference genomes (hg19 for human and mm9 for mouse), aggregated in different resolutions (1 Mbp, 500 kbp, and 100 kbp bins), and then normalized to correct for known biases using iterative correction and eigenvector decomposition (ICE) [21]. The data sets are deeply sequenced with 600–900 million reads, enabling us to examine both intra- and inter-chromosomal interactions. After binning and normalization, we had a total 2755, 5465, and 27,179 bins in human at 1-Mbp, 500-kbp, and 100-kbp resolution, respectively. In mouse, we had 2469, 4901, and 24,213 bins at 1-Mbp, 500-kbp, and 100-kbp resolution, respectively.

### Clustering algorithms for one data set

We considered three classes of clustering algorithms: hierarchical, *k*-means, and spectral clustering, each with five different distance metrics: Euclidean distance, Pearson’s correlation, Spearman’s correlation, contact counts, and log2 contact counts. We further adapted the distance to suit each method as described below.

#### Determining the number of clusters

We treat the number of clusters as an input parameter for the clustering algorithms examined. For our analysis, we inspected the interaction patterns obtained from spectral clustering. Specifically, we permuted the adjacency matrix to create a randomized graph, and compared the distribution of eigenvalues of the Laplacian of the original graph and the randomized graph. We observed that the difference in eigenvalues between the random and the original graph was not significant beyond the first 15, and therefore, we set 15 to be the upper limit on the number of clusters. We learned *k*∈2,5,10,15 clusters and manually inspected their contact count profiles and decided that *k*=10 provides the best results.

#### Hierarchical clustering

To perform hierarchical clustering with contact count and log2 of contact counts as distances, we subtracted the maximum value of the count (or log2 count) matrix from the given matrix. For the other three distance metrics, given the log2 of the genome-wide normalized contact count matrix, we calculated the distance of all pairs of bins using the pdist function in Matlab. To define the clusters, we used average linkage (using the linkage function) and the cluster function (with option maxclust set to *k*) to find *k* clusters. Using Euclidean distance and Spearman’s correlation, we observed a number of very small clusters (<10 elements) and one very large cluster. Because assessing statistical enrichment of signals is difficult for such small clusters, we applied a post-processing step to obtain more balanced clusters. Specifically, we kept partitioning the largest cluster until we reached *k* clusters with at least ten elements, and then added the clusters with less than ten elements to the largest cluster.

#### *k*-means

For Euclidean distance and Pearson’s correlation, we used the kmeans function in Matlab. This function does not provide clustering using Spearman’s correlation distance, so we implemented *k*-means with 1-Spearman’s correlation as the distance measure. To cluster with contact counts and log2 of contact counts, we also implemented a modified version of *k*-means similar to the *k*-means algorithm described in Yaffe et al. [19].

#### Spectral clustering

Our spectral clustering algorithm is motivated by the fact that the Hi-C interaction map can be viewed as a weighted graph with vertices representing regions. The weight of the edge between a pair of regions can correspond to the contact count between the two regions (or log2 of contact count) or a more indirect but global measure of similarity of the interaction profile of those regions (e.g. using a Spearman’s or Pearson’s correlation). A graph-based framework was recently shown to capture several topological properties of chromosome organization in yeast [22] and improve chromatin-mark-based genome annotation [23], suggesting that a graphical representation serves as a powerful representation for Hi-C data. Spectral clustering is a graph-clustering method that uses the eigenvectors of the Laplacian of a graph for clustering [24, 25, 43].

We used the algorithm described by Rohe et al. [24], which is based on clustering the eigenvectors corresponding to the largest eigenvalues of the graph Laplacian matrix. For each variant of similarity measure, we created a different weighted graph, with the weight representing the similarity measure. Let *A* denote an *n*×*n* adjacency matrix, where *n* is the total number of regions. Let *A*(*i*,*j*) denote the edge weight between regions *i* and *j*. For the Euclidean distance, *A*(*i*,*j*)=*M*−*e*_*i*,*j*_ where *e*_*i*,*j*_ is the Euclidean distance between row *i* and row *j* of the log2 of the normalized contact count matrix, and *M* is the maximum observed Euclidean distance. Thus, two regions that have a large value of *e*_*i*,*j*_ will be less similar to each other than two regions with a small value of *e*_*i*,*j*_. For the Pearson’s or Spearman’s correlation, *A*(*i*,*j*)=*c*_*i*,*j*_ if *c*_*i*,*j*_≥0, and *A*(*i*,*j*)=0 otherwise, where *c*_*i*,*j*_ is the correlation between row *i* and row *j* of the log2 of the normalized contact count matrix. For the normalized contact counts and log2 of normalized contact counts, *A*(*i*,*j*) was set to the corresponding count or log2 of the contact count between regions *i* and *j*. The Laplacian of the graph is defined as *L*=*D*^−1/2^*AD*^−1/2^ where *D* is a diagonal matrix with each element $D(i,i) = \sum _{k} a_{i,k}$. Thus, the Laplacian gives a normalized degree distribution of all vertices. We used the eigs function in Matlab to calculate the eigenvectors and eigenvalues of the Laplacian. Once we have the eigenvectors, the *k*-means algorithm is used to cluster the matrix **X**={*X*_1_,*X*_2_,…,*X*_*k*_} where *X*_*i*_ is a column vector in *R*^*n*^ and *X*_1_,*X*_2_,…,*X*_*k*_ are the first *k* eigenvectors of *L*, corresponding to the *k* largest eigenvalues of *L*.

### Description of cluster evaluation criteria

We used five different statistical measures to assess the quality of our clusters.

#### Davies–Bouldin index

We defined the DBI as

$$\text{DBI} = \frac{1}{k} \sum_{i=1}^{k} \text{max}_{j \neq i} D_{i,j} $$

where

$$D_{i,j} = \frac{\bar{d}_{i} + \bar{d}_{j}}{d_{i,j}}. $$

In the traditional definition of DBI, $\bar {d}_{i}$ is defined as the distance of elements in cluster *i* to its center, and *d*_*i*,*j*_ is the distance of the centers of clusters *i* and *j*. Because in some of our clustering methods we do not have a center for the clusters, we defined $\bar {d}_{i}$ as the average distance of all pairs of elements in cluster *i*, and *d*_*i*,*j*_ as the average distance of pairs of elements where one element was in cluster *i* and the second element was in cluster *j*. We used 1-Spearman’s correlation as the distance metric.

#### Silhouette index

We defined the SI as

$$\text{SI}=\frac{1}{k} \sum_{i=1}^{k} \frac{1}{\vert C_{i} \vert}\sum_{j \in C_{i}} s_{j} $$

where

$$s_{j} = \frac{b_{j} - a_{j}}{\text{max} \lbrace a_{j}, b_{j} \rbrace} $$

and *a*_*j*_ is defined as the average distance of element *j* to all other members of its own cluster (*C*_*i*_), and *b*_*j*_ is the average distance of element *j* to the members of the second best cluster (the cluster other than *C*_*i*_ with lowest average distance to element *j*). We used 1-Spearman’s correlation as the distance metric.

#### Delta contact counts

This measure was defined on the log of the contact count matrix. For each cluster *C*_*i*_, let in_*i*_ denote the average log of contact counts for pairs of regions in that cluster, and out_*i*_ denote the average log of contact counts for pairs of regions where one region is in cluster *C*_*i*_ and the other region is not. We define the delta contact count, *D*, as

$$D = \frac{1}{k} \sum_{i=1}^{k} \text{in}_{i} - \text{out}_{i}. $$

We expect that for a good cluster, the pairs of regions within the cluster should have higher contact counts. Therefore, the higher the value of *D*, the higher the quality of the clusters.

#### Number of enriched clusters

For each cluster and each genomic signal, we used the KS test to compare the distribution of the values of the given signal for the regions inside and outside the cluster. We test whether the values inside the cluster are significantly higher than values outside the cluster. If the *P* value returned by the KS test was lower than 0.05, we considered that cluster enriched for the given signal. We counted the number of clusters that were enriched for at least one signal. To calculate the *P* value of the KS test we used the kstest2 function of Matlab with the smaller switch.

#### ANOVA test

To test how well our clusters can separate the regulatory signals, we performed a one-way ANOVA test for each given signal and the cluster assignments for all regions examined. We used the anova1 function of Matlab, and used the sum of − log of *P* values over all the given signals to rank the clustering methods.

### Arboretum-Hi-C: a multi-task clustering approach for multiple Hi-C data sets

To perform multi-task clustering between the four cell lines, we first found a one-to-one mapping between orthologous 1-Mbp (and 500-kbp) bins between human and mouse and extracted contact count matrices corresponding to orthologous regions (see below). Next, we calculated the eigenvectors of the Laplacian as described above (spectral clustering with Spearman’s correlation). We ran Arboretum on the eigenvectors that had an orthologous region in the other species as described in detail below.

#### Orthology mapping between regions in human and mouse

To define the orthologous pairs of regions between human and mouse at a particular resolution *r*, we split the genome of each species into contiguous regions of *r* base pairs (1 Mbp or 500 kbp). We used a stringent criterion to define the orthology by requiring these regions to satisfy two filters. First, we used blastn with the option -evalue 1E-5 to align these regions to each other. For each pair of regions *h*_*i*_ and *m*_*j*_ from human and mouse, we sum the number of base pairs aligned between the two regions [ *A*(*h*_*i*_,*m*_*j*_)]. For a region *h*_*i*_ in human, we find the region from mouse *m*_*j*_ with the longest alignment to it: $m_{j} = \text {argmax}_{m_{j^{\prime }}} A(h_{i},m_{j^{\prime }})\phantom {\dot {i}\!}$. Similarly, for a region *m*_*j*_ in mouse, we find the region *h*_*i*_ in human with the longest alignment to it. We accept a pair of regions (*h*_*i*_,*m*_*j*_) as orthologous if they are reciprocal hits.

Our second filter used whole-genome alignments specified in chain files from the UCSC Genome Bioinformatics website (http://genome.ucsc.edu/) [44, 45]. We read the chain files and for each chain of alignments, we iterate over the alignment segments and add the length of the aligned segment to the corresponding pair of regions in human and mouse. For each region in human, we select the region in mouse with the largest sum of aligned segments (and vice versa for mouse to human) and selected the best reciprocal hits. We further filter these orthologous pairs by removing any pair with a sum of segments <0.1*r* (1 Mbp or 500 kbp). There is a significant agreement between the orthology mapping produced by the two approaches (∼95 % of the pairs produced from blastn are also in the other map). Our final set of orthologous mappings for input to Arboretum-Hi-C was obtained by taking the intersection of orthologous pairs from the above two filtering approaches. This results in 1318 orthologous regions between human and mouse at 1-Mbp resolution and 2342 regions at 500-kbp resolution.

#### Arboretum algorithm for multi-task clustering

Arboretum was developed to cluster multiple expression data sets, one from each species, while exploiting the gene and species tree phylogenies in the clustering using a probabilistic framework [30]. This approach favors orthologous genes having the same cluster assignment between species subject to the support in the data. Instead of clustering the expression of the genes, here we use Arboretum to cluster the eigenvectors of the Laplacian of the graphs produced from the Hi-C data; and rather than clustering the eigenvectors of each cell line separately, we cluster multiple Hi-C data sets simultaneously. To run Arboretum, we need a mapping between the elements that are being clustered (e.g. 1-Mbp regions) and also a tree structure to capture the relationships between data sets from different species and cell lines. We experimented with different tree structures and selected the one that gave us better likelihood (Additional file 1: Methods and Additional file 1: Figure S11).

#### Comparison of cluster similarity between pairs of cell lines/species

We used the hypergeometric test to compute the significance of similarity between pairs of clusters for each pair of cell lines/species. Given the matrix of − log10 of the hypergeometric test’s *P* value for pairs of clusters, the conservation score was defined as the sum of the diagonal elements (clusters with matched IDs) minus the sum of the off-diagonal elements (clusters with different IDs). Because independent spectral clustering does not provide a mapping of cluster assignments across data sets, we first used the Hungarian algorithm [46] to find the best one-to-one matching between the two given cluster assignments that maximizes the overlap between matched clusters, and using this matching we calculated the conservation score (as described above).

### Compartment identification and comparison to spectral clustering clusters

To define compartments, we followed the procedure described in Lieberman et al. [8]. We used the raw contact counts (before applying ICE for normalization) and calculated the genome-wide average contact count *I*_*s*_ for all possible genomic distances *s*. For each chromosome, we defined a matrix *M* by dividing the contact counts of pairs of regions at distance *s* by *I*_*s*_. We computed the Spearman’s correlation for entries in *M* and took the first principal component of this correlation matrix. We defined the two compartments based on positive and negative values of the first principal component.

To compare the spectral clusters to the two compartments in each chromosome, we used two different measures: the *F* score and the Rand index. To calculate the *F* score, we first count the number of pairs of regions that were in the same cluster in spectral clusters *s*, the number of pairs of regions that were in the same compartment *c*, and the number of pairs of regions that were grouped together in both methods *o*. We defined precision *p*=*o*/*s*, recall *r*=*o*/*c*, and *F* score

$$f= \frac{2 p r}{p+r}. $$

We defined the Rand index as

$$R = \frac{o+b}{\binom{n}{2}} $$

where *b* is the number of pairs of regions that were in different modules in both methods and *n* is the number of regions.

### One-dimensional genomic signals for interpretation of clusters

To interpret the clusters obtained by the different clustering methods examined, we obtained a number of genomic signals representing binding profiles of transcription factors, chromatin state, and density of genes and repeat elements. We aggregated these signals into fixed-size bins (1 Mbp or 500 kbp) or into variable-sized bins defined by TADs. Below we refer to both fixed- and variable-sized bins.

#### Number of genes

We downloaded the annotation files for hg19 and mm9 assembly from the Ensembl website [47]. We aggregated the genes in a bin and counted the number of genes in each and used these counts as a signal for each bin.

#### Transcription factors

We used the transcription factor narrow peak files from ENCODE [42] for CEBPB, CMYC, CTCF, JUND, MAFK, P300, POL2, POLR2A, RAD21, SMC3, TAF1, and TBP for hESCs, and CEBPB, CTCF, MAFK, POLR2A, and RAD21 for the IMR90 cell line. We aggregated the peaks in each bin and counted the number of peaks in each bin. The number of peaks per bin was used as a signal for the bin.

#### DNase I and histone marks

We used peak files from ENCODE [42] for DNase I, H3k4me1, H3k4me2, H3k4me3, H3k9ac, H3k9me3, H3k27ac, H3k27me3, H3K36me3, H3k79me2, and H4k20me1 in hESC, and DNase I, H3k4me1, H3k4me3, H3k9ac, H3k9me3, H3k27ac, H3k27me3, and H3k36me3 in mESC. We aggregated the peaks in a bin and counted the number of peaks in each bin and used these counts as features.

#### LADs and replication timing

We downloaded LADs from Meuleman et al. [28] and used the percentage of 1-Mbp bins covered with LADs as a feature. We also downloaded replication timing data from Ryba et al. [48] for hESC, and from Hiratani et al. [49] for mESCs, and used the average of the replication timing ratio (log2 of early to late) in each bin as a feature value. The LAD and replication timing data were aligned to hg18, and we used liftOver to map them to hg19 coordinates [44].

#### Other sequence features

We downloaded SINE, LINE, and LTR repeats from the UCSC Genome Bioinformatics website (http://genome.ucsc.edu/) [45]. For each type of repeat, we counted the number of repeats in each bin and used these counts as features.

### Availability of data and materials

The scripts, programs, and data sets used in this study are available at http://zenodo.org/record/49767 and https://bitbucket.org/roygroup/arboretum-hic (under GPLv3). The data sets supporting the conclusions of this article are included within the article (and its additional files).

## Ethics approval

Not applicable.

## Additional files

Additional file 1 Supplemental data including supplementary text, Figs. S1–S15 and Table S1. (PDF 8740 kb)

Additional file 2 The resulting cluster assignments reported in this study, and the feature matrices used for enrichment analysis. (XLS 4270 kb)
