# Supplementary material for: A multi-task graph-clustering approach for chromosome conformation capture data sets identifies conserved modules of chromosomal interactions
Source: Genome Biol. 2016 May 27;17:114. doi: 10.1186/s13059-016-0962-8 (PMC4882777; doi:10.1186/s13059-016-0962-8)
Supplement: Additional file 1 — Supplemental data including supplementary text, Figs. S1–S15 and Table S1. (PDF 8740 kb) [file 13059_2016_962_MOESM1_ESM.pdf]

# Supplementary Materials

## Contents

**1 Supplementary Methods** **2**

1.1 Comparing cluster assignments at different bin sizes and bin definitions . . . . . . . . . . . 2

1.2 Clustering TAD regions . . . . . . . . . . . . . . . . . . . . . . . . . . . . . . . . . . . . . . 3

1.3 Clustering inter-chromosomal (*trans*) interactions only . . . . . . . . . . . . . . . . . . . . 3

1.4 Statistical significance of cluster evaluation values to assess over-clustering . . . . . . . . . 4

1.5 Assessing the dependencies between different regulatory features . . . . . . . . . . . . . . . 5

1.6 Arboretum-HiC algorithm details . . . . . . . . . . . . . . . . . . . . . . . . . . . . . . . . 5

1.6.1 Initialization . . . . . . . . . . . . . . . . . . . . . . . . . . . . . . . . . . . . . 6

1.6.2 Learning the Arboretum-Hi-C model . . . . . . . . . . . . . . . . . . . . . . . . 6

1.6.3 Arboretum-Hi-C clusters at higher resolution . . . . . . . . . . . . . . . . . . . . 8

1.6.4 Arboretum-Hi-C clusters for inter-chromosomal interactions . . . . . . . . . . . . 8

1.6.5 The effect of alternative phylogenetic tree structure on Arboretum-Hi-C . . . . . . . 8

1.7 Assessing the ability of clustering methods to recover TADs . . . . . . . . . . . . . . . . . 9

1.8 Assessing ability of clustering methods to recover compartments . . . . . . . . . . . . . . . 9

**2 Supplementary Figures** **11**

**3 Supplementary Tables** **45**

**4 Supplementary Dataset** **45**

# 1 Supplementary Methods

## 1.1 Comparing cluster assignments at different bin sizes and bin definitions

Given two sets of cluster assignments, $C^1$ and $C^2$ of regions of potentially different lengths, our goal is to calculate the overlap of the two sets. Let $C_i^1$ denote the $i^{th}$ cluster of the first set of assignments and let $C_j^2$ denote the $j^{th}$ cluster from the second set of assignments. Note that there is no correspondence between $C_i^1$ and $C_i^2$. For every pair of clusters $C_i^1$ and $C_j^2$ define overlap between these clusters as

$$\text{overlap}(C_i^1, C_j^2) = \sum_{r \in C_i^1, r' \in C_j^2} \text{overlap}(r, r'),$$

where $r$ and $r'$ are regions and overlap$(r, r')$ is the number of base pairs that overlap between two regions. We map each $C_i^1$ to a cluster $C_{i^*}^2$ where $i^* = \arg\max_j$ overlap$(C_i^1, C_j^2)$, is the cluster in the second set with the largest overlap. We iterate over all clusters in the first cluster assignment and sum over the length of the best overlaps ($o_{12} = \sum_i$ overlap$(C_i^1, C_{i^*}^2)$). The overlap ratio is defined as $\frac{o_{12}}{l_1}$ where $o_{12}$ is the length of the overlap (base pair) and $l_1$ is the length of the genome covered by the first cluster assignment (bp). We calculate this ratio from $C^1$ to $C^2$ and vice versa. The similarity between two cluster assignments is then defined as the average of the two ratios. To test the significance of the computed overlap, we compared it to the overlap between a clustering to a random shuffling of itself. The overlap with the random clusters is dependent on the number of clusters and size of the clusters, but in all cases, overlap estimated by a random clustering is significantly lower than the estimated overlap between two estimated clusterings (**Supplementary Table 1**). For example the overlap observed between *trans* and *cis* and *trans* interactions of human ESC at 1Mbp resolution is 59%, while the overlap between the *cis* and *trans* clusters and a random shuffling of itself is 24$\pm$0.5%. To calculate the F-score between a pair of clusters $C_i^1$ and $C_j^2$ (shown in **Supplementary Figures S3, S7, S8, S12, S13**), we calculate the overlap of the two clusters as described before ($o =$ overlap$(C_i^1, C_j^2)$). We define the F-score as $\frac{2pr}{p+r}$ where $p = \frac{o}{len(C_i^1)}$ and $r = \frac{o}{len(C_i^1)}$ and $len(C_i^1)$ is the length of the cluster $C_i^1$.

## 1.2 Clustering TAD regions

We start by aggregating normalized contact counts at 40Kbp resolution in pairs of TAD regions. If $bin_i$ is inside $TAD_k$ and $bin_j$ is inside $TAD_l$, we add the normalized contact count of the pair $(bin_i, bin_j)$ to pair of TAD regions $(TAD_k, TAD_l)$. Because the length of TADs are variable (160Kb-5Mb), we normalized the aggregated sums by dividing by the size of the TADs; if the length $TAD_k$ is $l_k$ and $TAD_l$ is $l_l$, we divide the aggregated sum of $(TAD_k, TAD_l)$ by $l_k \times l_l$. We next clustered the matrix of the aggregated sums using spectral clustering (with Spearman correlation as the graph edge weights). **Figure S7** shows the resulting clusters. **Figure S7 a-e** shows the heatmap of Spearman correlation matrix of the aggregated sums, re-ordered by the spectral clusters, the distribution of chromosomes in clusters, patterns of enrichment of different regulatory signals, and the significance of the enrichment in the clusters (similar to **Figure 2**). We next compared the clustering of TADs to the clustering of fixed size bins at 1Mbp resolution and calculated their similarity (as described above). Clustering using TADs and 1Mbp bins have a 43% overlap, which is significantly higher than random. Furthermore, for 4 of the 10 clusters we can uniquely map the clusters from TADs to clusters using 1Mbp bins **Figure S7f**.

## 1.3 Clustering inter-chromosomal (*trans*) interactions only

To create the adjacency matrix, $A$ using inter-chromosomal interactions only, we calculated the Spearman correlation of interaction profile of regions, after excluding the intra-chromosomal (*cis*) interactions. For a pair of regions $r_1$ and $r_2$, where $r_1$ is in chromosome $i$ and $r_2$ is in chromosome $j$, we define their trans correlation, $\text{corr}_{trans}(r_1, r_2)$, as the Spearman correlation between two vectors $x_{r_1}$ and $x_{r_2}$. Each entry $x_{r_1}(t)$ corresponds to all regions $t$ that are not in chromosomes $i$ or $j$. Each entry of the adjacency matrix is then defined as

$$A_{r_1,r_2} = \begin{cases} 0 & \text{if } chr(r_1) = chr(r_2) \\ 0 & \text{if } corr_{trans}(r_1, r_2) < 0 \\ corr_{trans}(r_1, r_2) & \text{otherwise} \end{cases}$$

where $chr(r)$ indicates the chromosome of region $r$. We then perform spectral clustering on $A$ as described before. **Supplementary Figure S3a,b,c** shows the heatmap of the adjacency matrix, and the

enrichment of different regulatory signals in these clusters for human ES cell line. We find that these clusters exhibit similar patterns of enrichment as the clusters produced when using both inter and intra-chromosomal interactions (**Figure 2**). **Supplementary Figure S3d, e** shows the distribution of chromosome in clusters, and the distribution of clusters in chromosomes. 6 out of 10 clusters consist of regions from different chromosomes, 3 of these 6 are enriched with different regulatory signals associated with open chromatin, and 3 are enriched for LADs and are associated with closed chromatin state. We also observe that all chromosomes are split into more than 1 cluster. **Supplementary Figure S3f** shows the overlap of these clusters to clusters obtained using both inter and intra chromosomal interactions. We observe significant overlap (59%) between the two clusterings, suggesting that at this resolution, we can recover similar structures from both *trans* and *cis* and *trans* interactions.

### 1.4 Statistical significance of cluster evaluation values to assess over-clustering

Because several clusters exhibited similar patterns of enrichment that could be grouped into high and low activity regions, we tested whether $k = 10$ was overclustering the data, by computing the expected value of the evaluation criteria in a random setting. Specifically, to evaluate whether the scores that we observe from different evaluation metrics are significant, we compare these scores to random cluster assignments (**Supplementary Figure S5**). We generated 100 random cluster assignments by shuffling the cluster assignments from spectral (Spearman$>$ 0) clustering. Davies-Bouldin index, Delta-contact counts, and silhouette index for the actual clustering result is significantly better (red star in **Supplementary Figure S5**) than random cluster assignments. As an additional sanity check of our clustering, we compared the distribution of interaction strengths within and between clusters for every pair of clusters (**Supplementary Figure S5b, c**). **Supplementary Figure S5b** shows the distribution of positive Spearman correlation within regions of clusters C0 and C1, and between regions in clusters C0 and C1. We observe that regions in each cluster are significantly more correlated to themselves compared to regions between two clusters. **Supplementary Figure S5c** shows the the distribution of correlation between and within clusters, for all pairs of clusters. We observe significant difference in between and within correlations for all pairs of clusters (KS-test $p$-value $< 1E-20$).

## 1.5 Assessing the dependencies between different regulatory features

The regulatory genomic signals that we used for assessing enrichment in the clusters were highly correlated (**Supplementary Figure S4a, b**). Because previously open chromatin assays measured through DNase I footprinting has been a strong predictor of interacting and non-interacting regions, it is possible that the enrichment patterns that we observed were a consequence of their association of DNase I, and not due to the clustering of the Hi-C data. To test whether the clustering is providing additional information over DNase I for the other non-DNase I features we computed the conditional mutual information between two random variables, given the DNase I signal. One random variable represented the cluster partition and the second random variable represented one of the non-DNase I signals. Before calculating the conditional mutual information, we first discretize each feature by clustering it into 3 clusters (using Matlab's `kmeans`). To assess the significance of the conditional mutual information for a feature and a clustering, we performed a permutation test. We shuffled the cluster assignments for 1000 times and calculated mean and STD of the mutual information values for the 1000 random cluster assignments. We calculated the significance of the conditional mutual information by comparing the actual value to the mean and STD from random permutations (using Matlab's `ztest`). **Supplementary Figure S4c-g** shows the conditional mutual information of different features and the results of different spectral clustering methods, given the DNaseI feature vector, and the corresponding random value. Even though different regulatory features are significantly correlated, we observe that the enrichment of these features in the resulting clusters can not be explained using DNase I alone and the clusters are providing additional information.

## 1.6 Arboretum-HiC algorithm details

Given the Hi-C contact count matrices of $M$ different cell line/species, and a mapping between the regions in these cell lines and/or species, the goal of Arboretum-Hi-C is to cluster the contact counts. Between cell lines of the same species, the regions are identical. Between two species, we define the mapping using orthology of the genomic bins (as described in the manuscript methods). The original Arboretum algorithm was developed to cluster multiple expression clusters, one for each species. Below we describe the key differences in the initialization and learning algorithm due to the nature of the Hi-C data.

### 1.6.1 Initialization

To cluster Hi-C contact count matrices using the spectral clustering algorithm, we computed the top $K$ eigenvectors of the graph Laplacian and apply Gaussian mixture model on each set of eigenvectors. Here $K$ denotes the number of clusters in each dataset. Let $\mathbf{X}_i$ denote the set $n \times k$ matrix of the $K$ largest eigenvectors where each column corresponds to an eigenvector. We merge this matrix into $\mathcal{X} = [\mathbf{X}_1, \cdots, \mathbf{X}_M]$. We next applied the $k$-means algorithm to this merged matrix with 50 random restarts and pick the clustering with the best objective. These cluster assignments are projected onto each Hi-C dataset to estimate the initial means and diagonal covariance elements of each Gaussian mixture. There is one mixture per leaf node.

### 1.6.2 Learning the Arboretum-Hi-C model

The parameters of Arboretum-Hi-C are the transition matrices for each branch in the tree, the prior probability of each cluster, and the Gaussian mixture model associated with each leaf node which has the measured dataset. As in Arboretum, we use an Expectation Maximization (EM) framework to cluster the data: in the E step the hidden cluster assignments are inferred, and in the M step the Gaussian mixture model parameters and the transition parameters are estimated. The EM algorithm is very similar to the original Arboretum algorithm, with the key difference that we are applying it to eigenvectors rather than expression values.

**Expectation:** Let $z_{ri}^{k}$ denote the indicator variable that region $i$ in the last common ancestor, $r$ (the root of the phylogenetic tree) is in cluster $k$. Let $z_{ti}^{k|k'}$ denote the indicator variable that region $i$ is assigned to module $k$ in node $t$, while in its immediate ancestor it was assigned to $k'$. Let $\gamma_{ti}^{k|k'}$ be the posterior probability of this event. Let $P_t(k|k')$ denote the transition probability of a region to switch to cluster $k$ given that it was in cluster $k'$ in $t$'s immediate ancestor. We have a $P_t(k|k')$ for each branch of the tree. Let $\{\mu_1^t, \cdots, \mu_k^t, \Sigma_1^t, \cdots, \Sigma_k^t)\}$ denote the mixture Gaussian parameters when $t$ corresponds to a leaf node. We assume that module assignment of region $i$ in node $t$ is only dependent on the sub-tree rooted at $t$. Given this independent assumption, we can infer these posterior probabilities in a recursive manner. We start at a leaf node $t$ and estimate the $\gamma$ variables based on the row, $x_i^t$ of the eigenvector matrix $\mathbf{X}_t$ corresponding to region $i$ and the Gaussian mixture parameters with this node:

$$\gamma_{ti}^{k|k'} = P_t(k|k')P(x_i^t|\mu_k^t, \Sigma_k^t)$$

$P(x_i^t|\mu_k^t, \sigma_k^t)$ is the probability of observing $x_i^t$ from a Gaussian with parameters $\mu_k^t$ and $\Sigma_k^t$. For each internal node $\gamma$ is estimated as:

$$\gamma_{ti}^{k|k'} = P_t(k|k')n^{left(t)}(k)n^{right(t)}(k)$$

where $left(t)$ and $right(t)$ are respectively left child and right child of node $t$, and $n^t(k) = \sum_{k''} \gamma_{ti}^{k''|k}$ is the normalization term. In other words, $n^{left(t)}(k)$ and $n^{right(t)}(k)$ sum over all possible cluster assignments of region $i$ in children of $t$.

**Maximization:** The maximization step of the inference is very similar to standard Gaussian mixture models. For module $k$ in node $t$ we have:

$$\mu_{tk} = \frac{\sum_i \sum_{k'=1}^{K} \gamma_t^{k,k'} x_i^t}{\sum_i \sum_{k'=1}^{K} \gamma_t^{k,k'}}$$

where $\gamma_t^{k,k'}$ is the expected value of joint assignment to module $k$ in $t$ and to module $k'$ in immediate ancestor of $t$. The covariance matrix can be estimated similarly.

**Likelihood:** The penalized likelihood of the model is defined as the data likelihood (the combination of likelihood of mixture of Gaussians in leaf nodes and transition probabilities from parent nodes to child nodes) penalized by the number of parameters: $L - \frac{n_p}{2log(n_o)}$ where $L$ is the data likelihood, $n_p$ is the number of free parameters, and $n_o$ is the number of regions. For a simple mixture of Gaussian the number of parameters would be $2KT$ ($K$ clusters with a mean and diagonal covariance matrix with $T$ elements corresponding to $T$ measurements in the input data). We note that in the spectral clustering framework, $K = T$ and therefore the number of parameters is $2K^2$. In Arboretum additionally we will have $K(K-1)$ parameters for the transition matrices in leaf nodes and internal nodes, and $K - 1$ parameters in root node for the initial prior probabilities of the modules. So the number of parameters in the complete model will add to $n_p = (2K^2 + K(K-1))S_e + (K(K-1))S_a + (K-1)$ where $S_e$ correspond to the number of leaf

nodes (cell line/species) and $S_a$ correspond to the number of internal nodes excluding the root node.

#### 1.6.3 Arboretum-Hi-C clusters at higher resolution

We applied Arboretum-Hi-C to both regions of 1Mbp and 500Kbp size. **Supplementary Figure S12a,b** show the results of Arboretum-Hi-C at 500Kbp resolution. Normalized contact counts were aggregated at 500Kbp resolution. We defined the orthology map between the 500Kbp regions as described before for 1Mbp resolution maps. We observe similar patterns of enrichment as we observed in the 1Mbp resolution clusters. **Supplementary Figure S12c-e** shows the percentage of overlap between clusters recovered at 500Kbp and 1Mbp resolutions, and we observe significant overlap between the clusters in all cell lines.

#### 1.6.4 Arboretum-Hi-C clusters for inter-chromosomal interactions

We also applied Arboretum-Hi-C to *trans*-only interactions in a manner similar to the single task spectral clustering, but excluding any interaction that is in *cis* in either human or mouse. We considered only those regions that have an ortholog in human and mouse. As before, to define the *trans* correlation of two regions, $r_1$ and $r_2$, we first exclude all regions, $r'$ that are in the same chromosome as $r_1$ or $r_2$ or if $r'$'s ortholog is in the same chromosome as $r_1$'s ortholog or $r_2$'s ortholog in the other species. We compute the Spearman correlation between these two vectors, $corr_{trans}(r_1, r_2)$.

We then define our adjacency matrix as:

$$A_{r_1,r_2} = \begin{cases} 0 & \text{if } chr_h(r_1) = chr_h(r_2) \vee chr_m(r_1) = chr_m(r_2) \\ 0 & \text{if } corr_{trans}(r_1, r_2) < 0 \\ corr_{trans}(r_1, r_2) & \text{otherwise} \end{cases}$$

$chr_h(r)$ and $chr_m(r)$ are the human and mouse chromosomes of region $r$.

#### 1.6.5 The effect of alternative phylogenetic tree structure on Arboretum-Hi-C

To test the effect of the structure of the phylogenetic tree on the results of Arboretum-Hi-C, we tested two alternative tree structures, one where the cell lines from the same species are siblings (tree1) and one where ES cell lines are siblings (tree2, **Supplementary Figure S11a**). We observed that the likelihood of the

model is significantly higher when using tree1 (**Supplementary Figure S11b**). We furthermore investigated the conservation scores between the pairs of cell lines when using different structures of phylogenetic tree (**Supplementary Figure S11c**). We found that even though using tree2 structure decreases the within species conservation scores (hESC vs. hIMR90 and mESC vs. mCortex), these scores are still significantly higher than the between species conservation scores, suggesting that for these cell lines, Hi-C data from the same species are more similar to each other than the Hi-C datasets from matched cellular stages.

### 1.7 Assessing the ability of clustering methods to recover TADs

We examined the ability of spectral and other clustering methods to recover TADs. We first examined a small portion of chromosome 1 of mESC (3Mb-9.5Mb at 40Kbp resolution). **Supplementary Figure S10a** shows the log2 of contact count for these regions, and **Supplementary Figure S10b** highlights the clusters recovered by different methods. Even though there are some disagreement between the clusters from different methods, we observe significant overlap between these clusters and the reported TADs. These results suggest that clustering could be a useful approach in recovering different topological structures.

**Supplementary Figure S10c** shows a graph representation of these regions. Edges correspond to top 10% of contact counts between the regions, and nodes are coloured to highlight different TADs (as reported by Dixon et al. [1]). We repeated our TAD analysis for all the chromosomes by extracting the contact count matrix corresponding to the reported TADs in each chromosome, and clustering this matrix using the different methods ($k$ = number of TADs in the chromosome). Because using global Spearman correlation can wash out the correlation information associated with the smaller bin size of 40Kbp, here we only used contact counts and log2 of contact counts. **Supplementary Figure S10d** shows the percentage overlap of different method and the TADs. We observe significant agreement between different clustering methods and the reported TADs in all chromosomes.

### 1.8 Assessing ability of clustering methods to recover compartments

To assess the ability of clustering methods in recovering compartments, we first applied our different clustering method on the chromosome 1 of hESC. To define the compartments, we used the procedure described in Lieberman-Aiden et al. [2]. The raw contact counts between regions $r_1$ and $r_2$ were normalized by divid-

ing it by the average contact count at distance of $dist(r_1, r_2)$. Different clustering methods were applied to this normalized matrix, and compartments were defined by positive and negative portions of the first principal component of the Spearman correlation of the normalized contact count. **Supplementary Figure S10e** shows the heatmap of the Spearman correlation of the normalized matrix, and **Supplementary Figure S10f** highlights the clusters recovered by different methods. We observe that most of the clusterings have significant overlap with the compartments, though the methods that use correlations distance (Spearman or Pearson) have better success in recovering the compartments, which is expected since the compartments were defined using the correlation of the normalized contact count matrix. We repeated our clustering for all the chromosomes in the human ESC dataset and compared the overlap of compartments to clusters obtained from different clustering methods. Because clustering of Spearman correlation had the best agreement with the compartments in chromosome 1, here we only compared different clustering methods using Spearman correlation as similarity measure. **Supplementary Figure S10g** shows the percentage overlap of different method and the compartments. We observe significant agreement between different clustering methods and the recovered compartments in all chromosomes.

# 2 Supplementary Figures

## List of Figures

S1 Spectral clustering using different distance measures . . . . . 14
S2 Hierarchical clustering using different distance measures . . . . . 18
S3 Clusters using inter-chromosomal (*trans*) interactions only . . . . . 20
S4 Assessing conditional mutual information between regulatory genomics signals . . . . . 22
S5 Distribution of cluster evaluation measures in random cluster assignments . . . . . 24
S6 Spectral clustering and analysis of mouse ESC Hi-C data . . . . . 26
S7 Clustering of TADs and comparison to fixed size bins. . . . . . 28
S8 Spectral clustering at different resolutions . . . . . 30
S9 Comparison of compartments to spectral clusters . . . . . 32
S10 Comparison of TAD like structures and TADs . . . . . 34
S11 Different phylogenetic/hierarchical relationships between datasets . . . . . 36
S12 Arboretum-Hi-C at different resolutions . . . . . 38
S13 Multi-task clustering using inter-chromosomal interactions only . . . . . 40
S14 Identifying signals associated with divergent clusters across species . . . . . 42
S15 Differential enrichment of measured TFs in regions that switch module assignment across tissues . . . . . 44

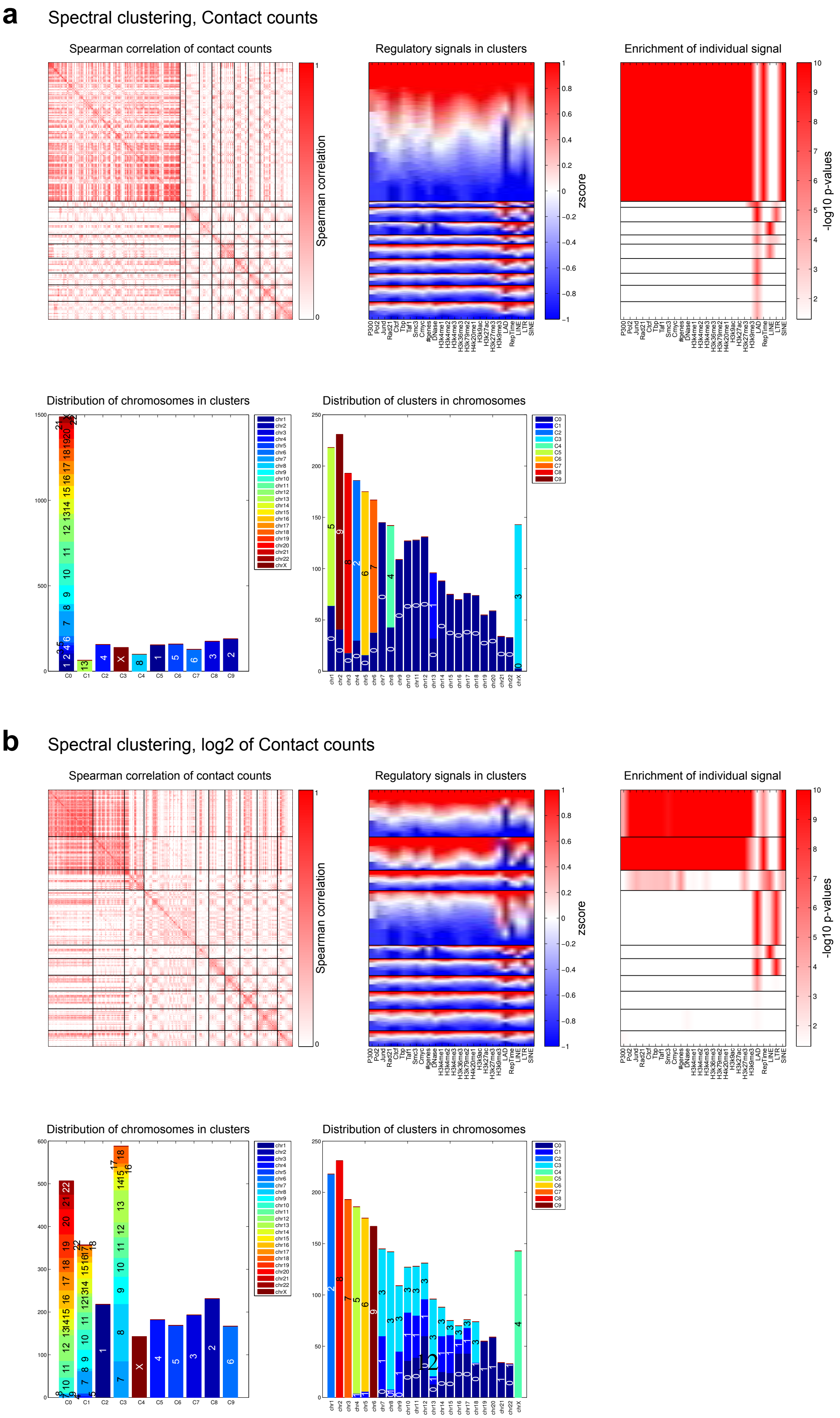
a
Spectral clustering, Contact counts
Spearman correlation of contact counts
Spearman correlation
Regulatory signals in clusters
zscore
Enrichment of individual signal
-log10 p-values
P300
Pol2
Jund
Rad21
Ctcf
Tbp
Taf1
Smc3
Cmyc
#genes
DNase
H3k4me1
H3k4me2
H3k4me3
H3k36me3
H3k79me2
H4k20me1
H3k9ac
H3k27ac
H3k27me3
H3k9me3
LAD
RepTime
LINE
LTR
SINE
Distribution of chromosomes in clusters
C0
C1
C2
C3
C4
C5
C6
C7
C8
C9
Distribution of clusters in chromosomes
chr1
chr2
chr3
chr4
chr5
chr6
chr7
chr8
chr9
chr10
chr11
chr12
chr13
chr14
chr15
chr16
chr17
chr18
chr19
chr20
chr21
chr22
chrX
b
Spectral clustering, log2 of Contact counts
Spearman correlation of contact counts
Spearman correlation
Regulatory signals in clusters
zscore
Enrichment of individual signal
-log10 p-values
Distribution of chromosomes in clusters
Distribution of clusters in chromosomes

**c** Spectral clustering, Euclidean distance

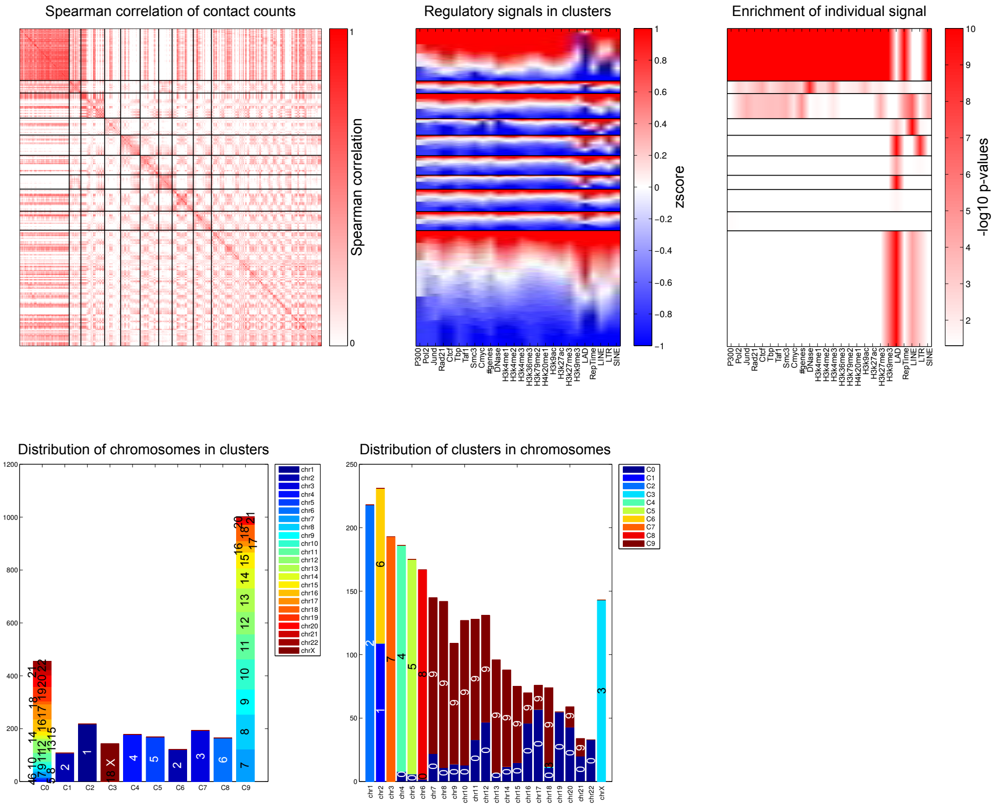


**d** Spectral clustering, Pearson correlation distance

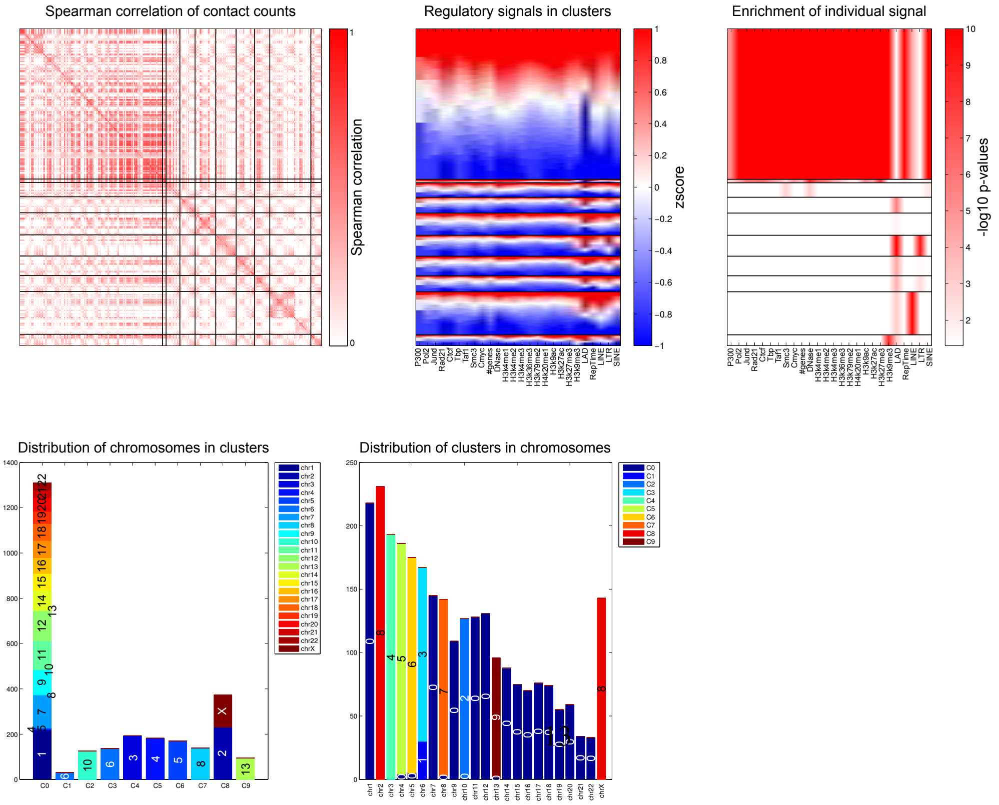

Figure S1: Spectral clustering of the human ESC Hi-C data using four different distance metrics: **(a)** Contact counts, **(b)** log2 of contact counts, **(c)** Euclidean distance, and **(d)** Pearson correlation distance. Figures in panels **a**-**d** follow the same legend as **Figure 2**. In the first row of each panel, the first plot on the left is the heatmap of Spearman correlation matrix of contact counts, reordered according to the spectral cluster assignments. The second plot is the enrichment of different regulatory features reordered according to the cluster assignments. The third plot on the right is -log10 of KS-test $p$-values. The first plot in the second row is the distribution of chromosomes in each cluster and the last plot is the distribution of clusters in each chromosome.

a Hierarchical clustering, Pearson correlation distance

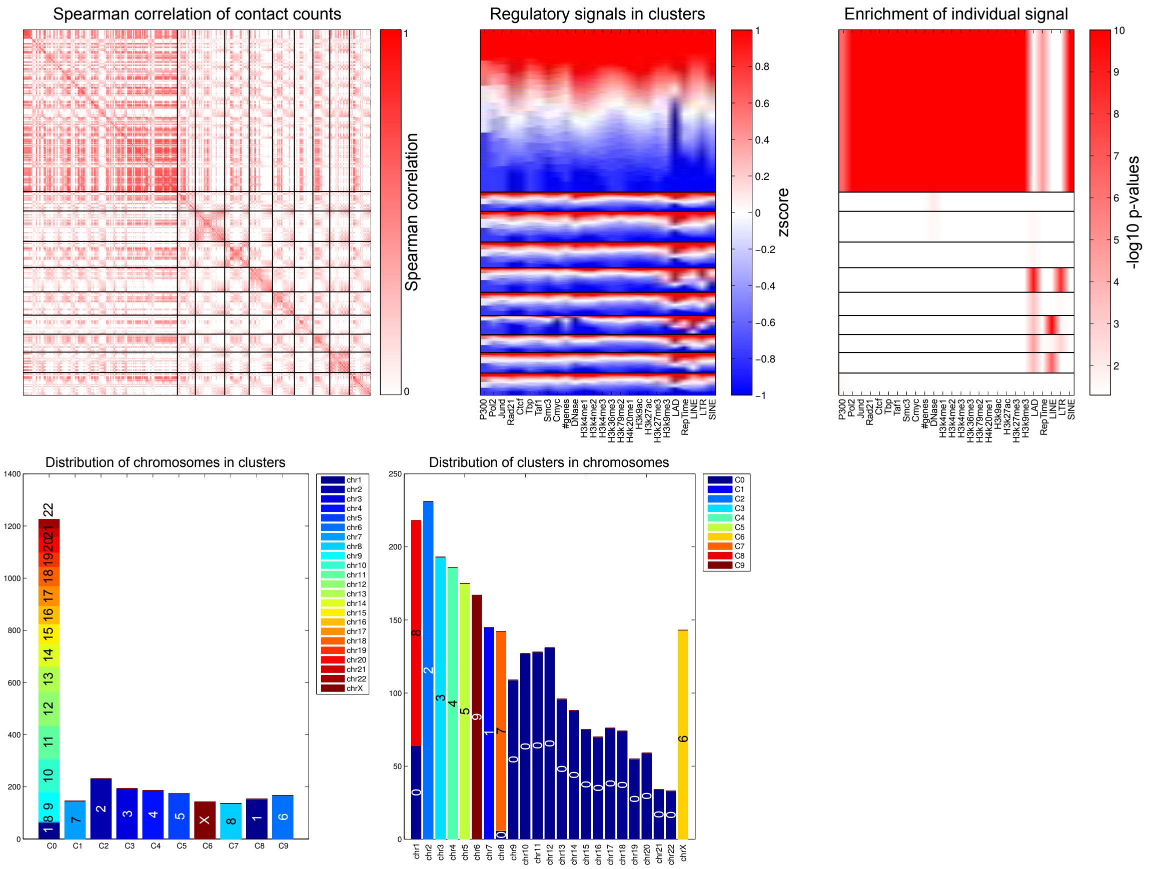


b Hierarchical clustering, Euclidean distance

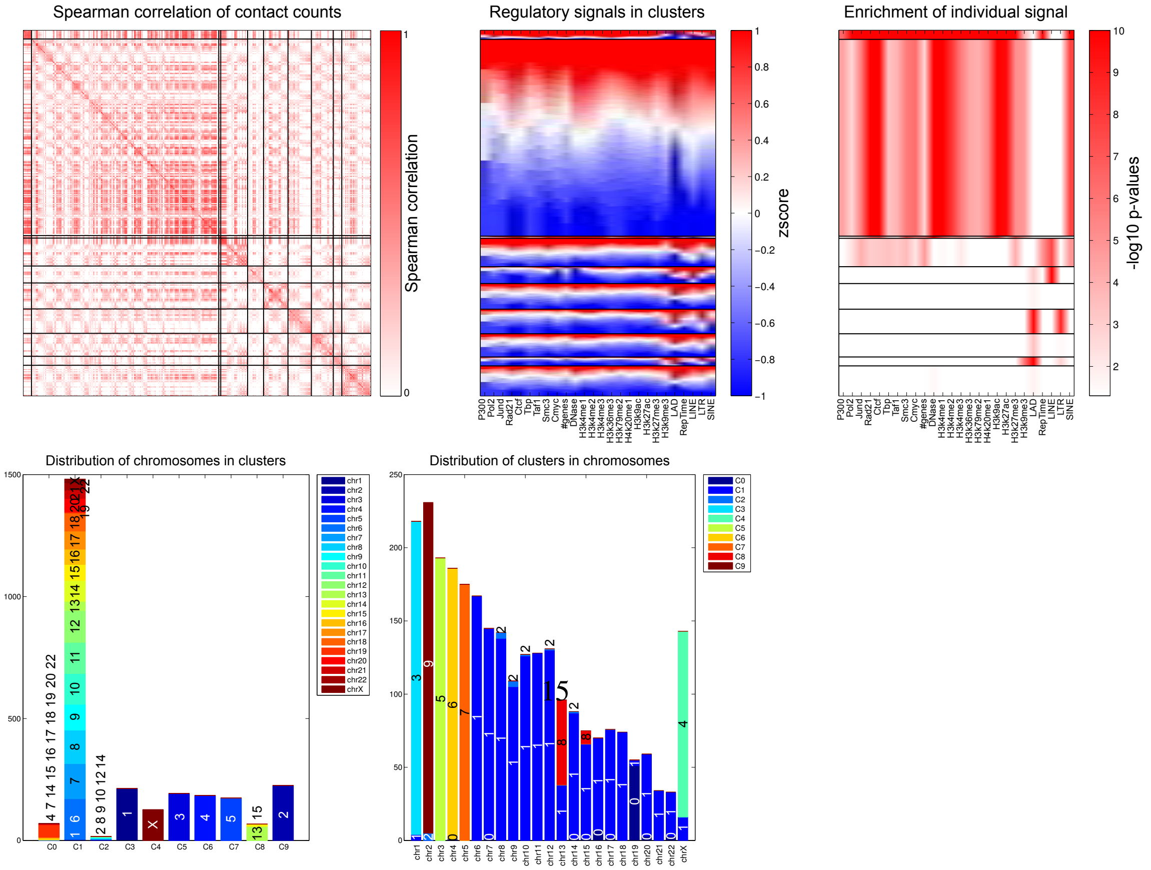

**c** Hierarchical clustering, Spearman correlation distance

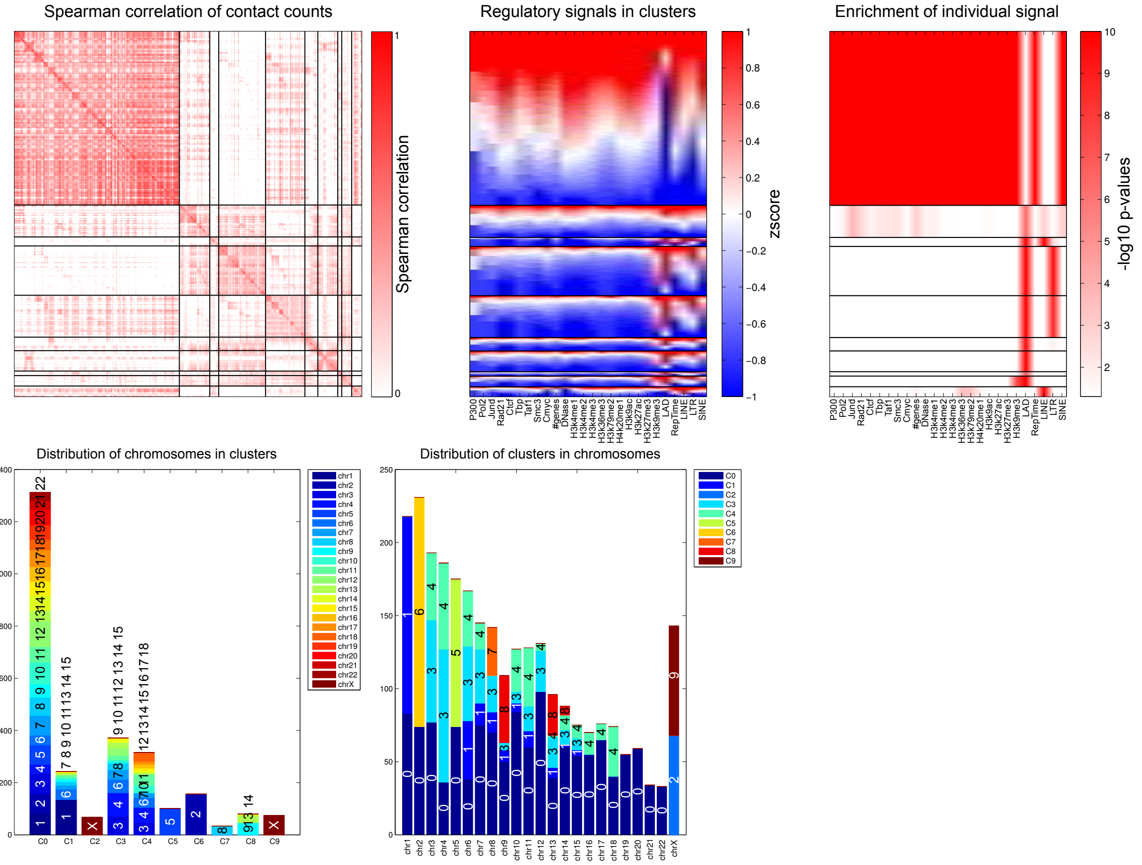


**d** Hierarchical clustering, Contact counts distance

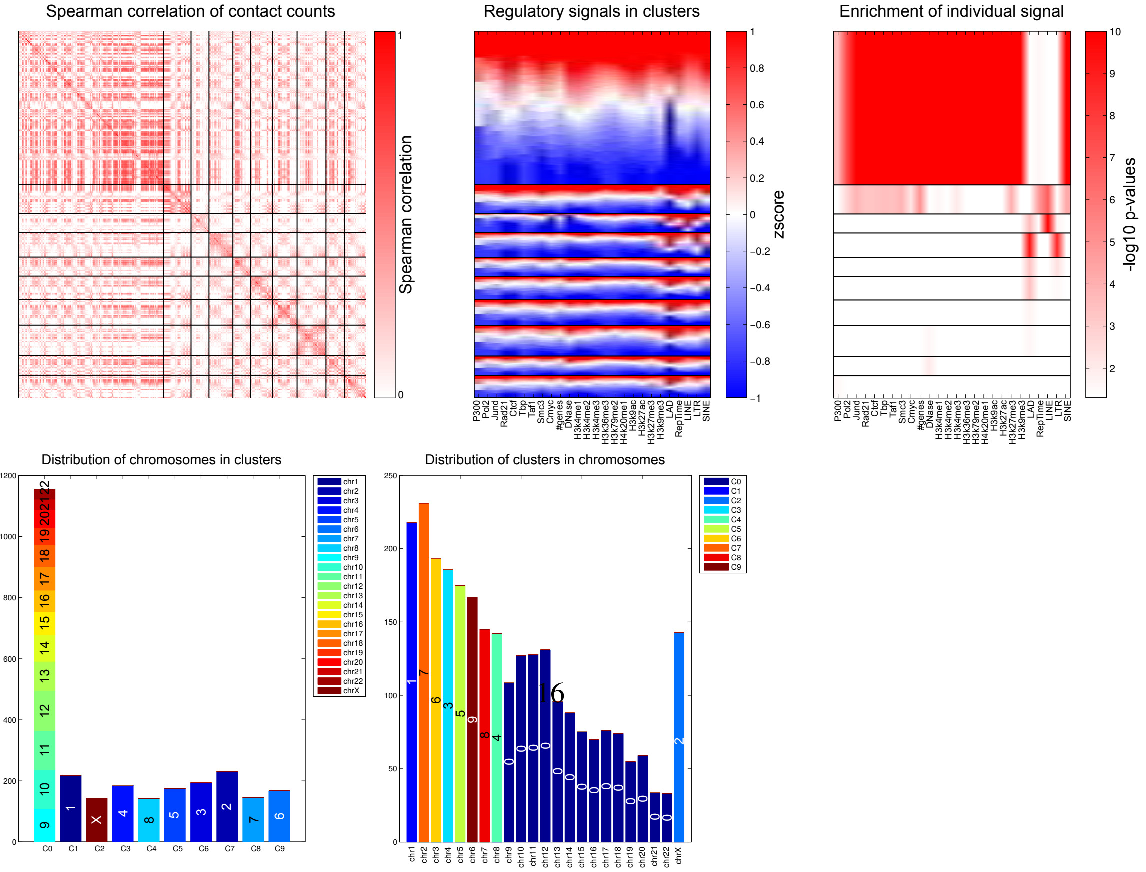

**e** Hierarchical clustering, log2 Contact counts distance

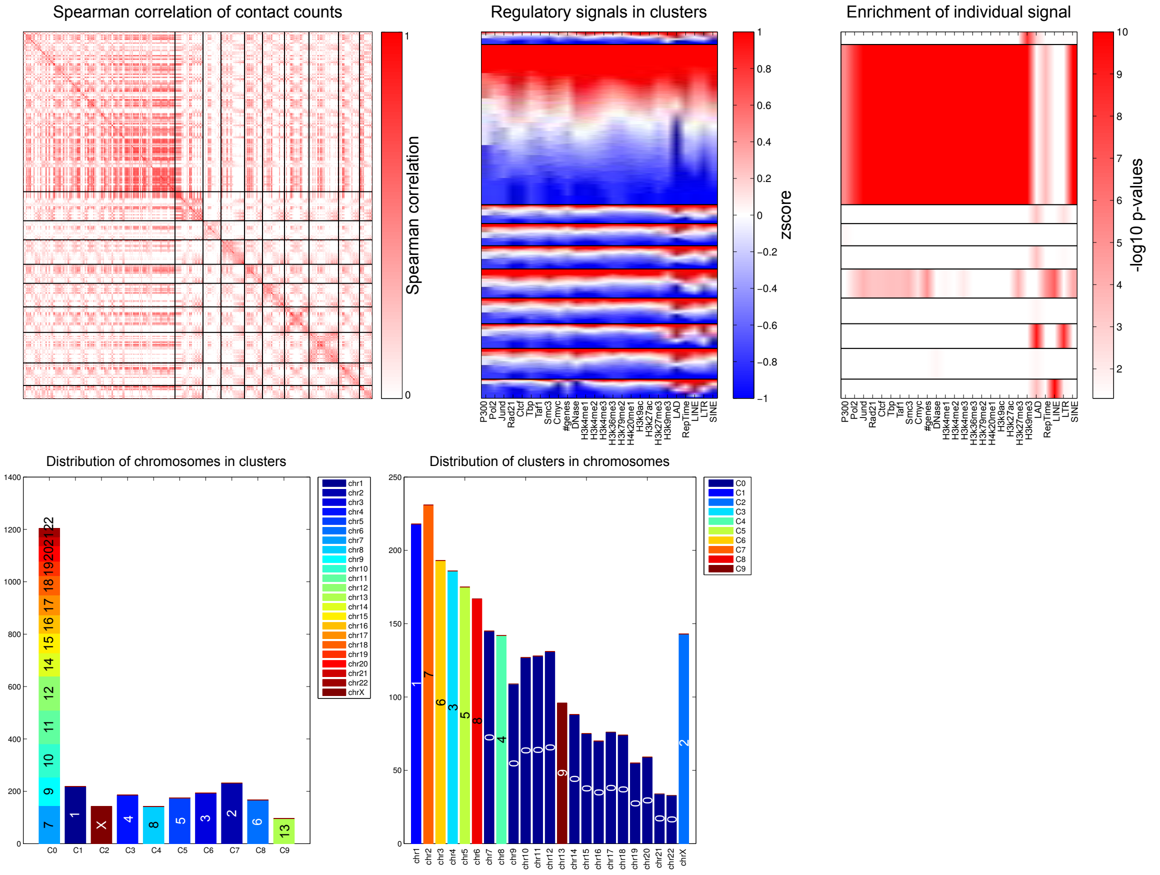

Figure S2: Hierarchical clustering of the human ESC Hi-C data using five different distance metrics: **(a)** Pearson correlation, **(b)** Euclidean distance, **(c)** Spearman correlation, **(d)** Contact counts, and **(e)** log2 of Contact counts. Each panel, **a**-**e**, follows the same legend as **Figure 2**. In the first row of each panel, the leftmost plot is the heatmap of Spearman correlation matrix of contact counts, reordered according to the spectral cluster assignments. The second plot is the enrichment of different regulatory features reordered according to the cluster assignments. The rightmost plot is the -log10 of KS-test $p$-values. The first plot in the second row is the distribution of chromosomes in each cluster and the last plot is the distribution of clusters in each chromosome.

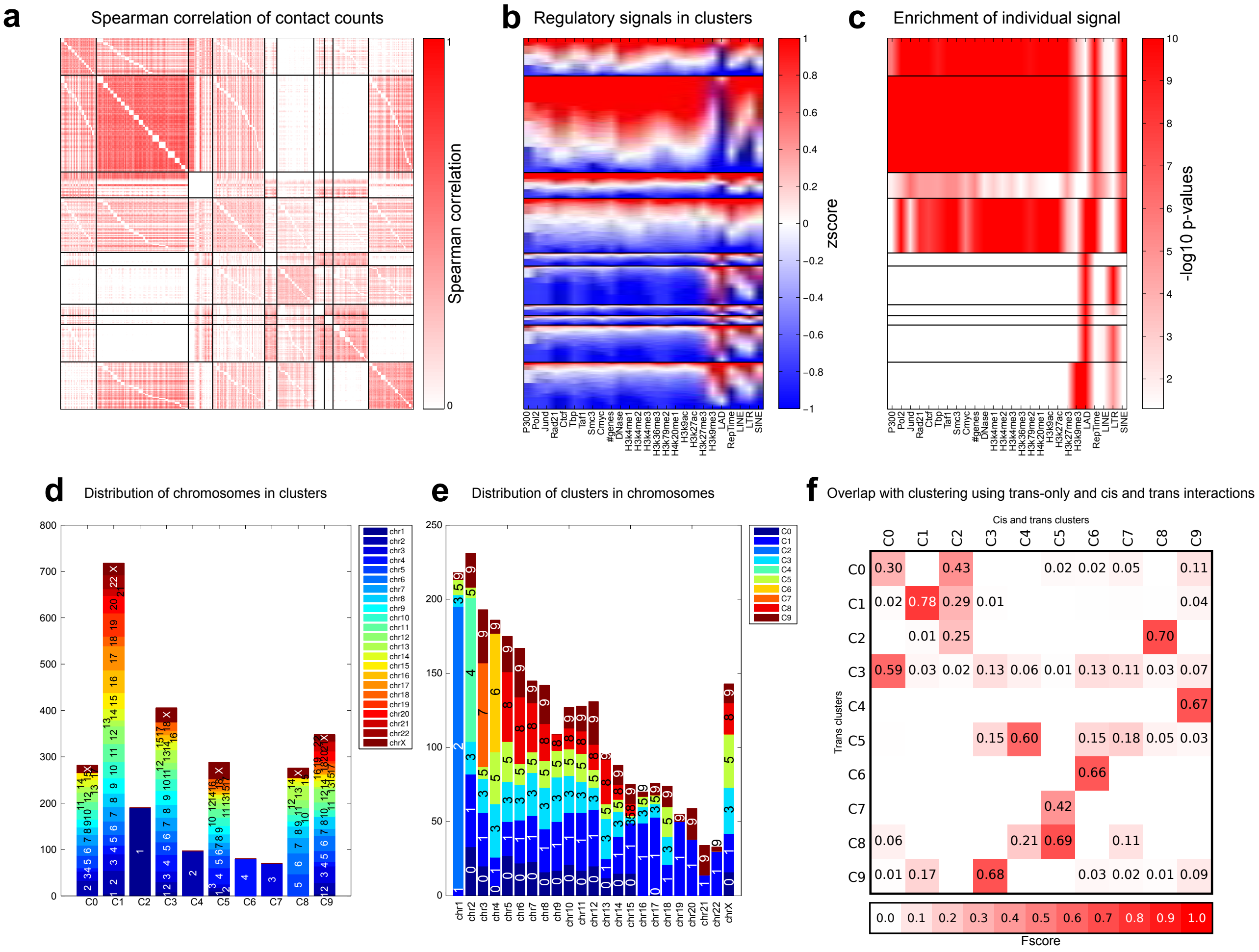

a
Spearman correlation of contact counts
Spearman correlation
b
Regulatory signals in clusters
zscore
c
Enrichment of individual signal
-log10 p-values
P300
Pol2
Jund
Rad21
Ctcf
Tbp
Taf1
Smc3
Cmyc
#genes
DNase
H3k4me1
H3k4me2
H3k4me3
H3k36me3
H3k79me2
H4k20me1
H3k9ac
H3k27ac
H3k27me3
H3k9me3
LAD
RepTime
LINE
LTR
SINE
d
Distribution of chromosomes in clusters
C0
C1
C2
C3
C4
C5
C6
C7
C8
C9
e
Distribution of clusters in chromosomes
chr1
chr2
chr3
chr4
chr5
chr6
chr7
chr8
chr9
chr10
chr11
chr12
chr13
chr14
chr15
chr16
chr17
chr18
chr19
chr20
chr21
chr22
chrX
f
Overlap with clustering using trans-only and cis and trans interactions
Cis and trans clusters
Trans clusters
C0 0.30 0.43 0.02 0.02 0.05 0.11
C1 0.02 0.78 0.29 0.01 0.04
C2 0.01 0.25 0.70
C3 0.59 0.03 0.02 0.13 0.06 0.01 0.13 0.11 0.03 0.07
C4 0.67
C5 0.15 0.60 0.15 0.18 0.05 0.03
C6 0.66
C7 0.42
C8 0.06 0.21 0.69 0.11
C9 0.01 0.17 0.68 0.03 0.02 0.01 0.09
0.0 0.1 0.2 0.3 0.4 0.5 0.6 0.7 0.8 0.9 1.0
Fscore

Figure S3: Spectral clustering on human ESC Hi-C data using inter-chromosomal interactions only. **(a)** Shown is the heatmap of Spearman correlation matrix of contact counts between regions ordered by the cluster assignment. **(b)** Different regulatory features grouped according to the spectral cluster assignments. **(c)** -log10 of KS-test $p$-values for each regulatory feature enrichment in each cluster. **(d)** The bar plot shows the distribution of chromosomes in the clusters. **(e)** The bar plot shows the distribution of clusters in the chromosomes. **(f)** Overlap of the clusters inferred from *trans*-only interactions and using *cis* and *trans* interactions.

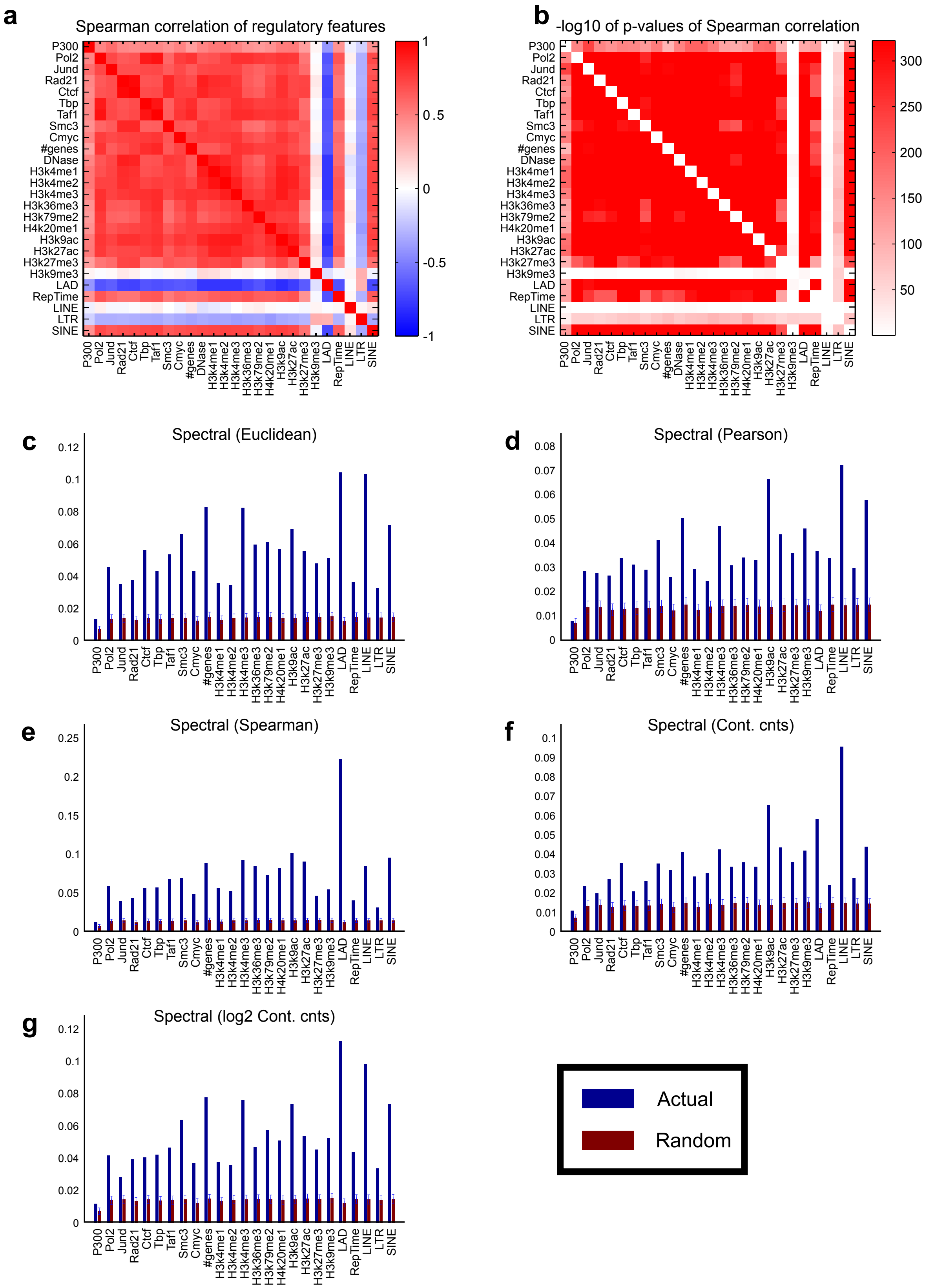
a
Spearman correlation of regulatory features
b
-log10 of p-values of Spearman correlation
c
Spectral (Euclidean)
d
Spectral (Pearson)
e
Spectral (Spearman)
f
Spectral (Cont. cnts)
g
Spectral (log2 Cont. cnts)
Actual
Random

Figure S4: Assessing dependencies between regulatory features and information gain due to spectral clustering. **(a)** Spearman correlation of different regulatory features at 1Mbp resolution. **(b)** The -log10 of $p$-values corresponding the the correlation values from panel **a**. (**c**-**g**) Conditional mutual information of a regulatory feature and cluster assignments given the DNase I feature. Each bar plot shows the conditional mutual information for the actual inferred clusters (blue) and the average conditional mutual information for 1000 randomized clusters (red). Error bars correspond to standard deviation.

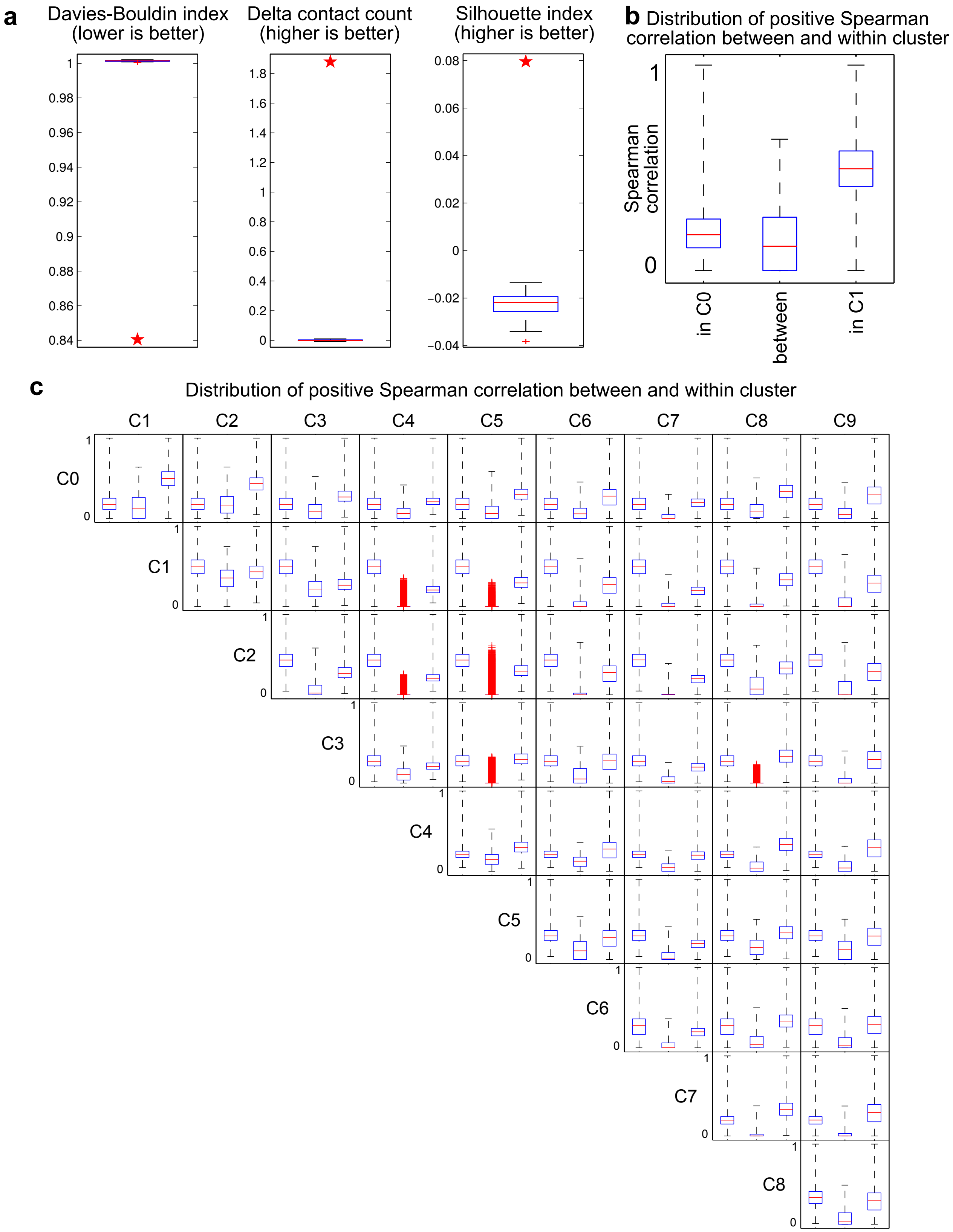
a
Davies-Bouldin index
(lower is better)
Delta contact count
(higher is better)
Silhouette index
(higher is better)
b Distribution of positive Spearman
correlation between and within cluster
Spearman
correlation
in C0
between
in C1
c
Distribution of positive Spearman correlation between and within cluster
C1
C2
C3
C4
C5
C6
C7
C8
C9
C0
C1
C2
C3
C4
C5
C6
C7
C8

Figure S5: Statistical significance of the cluster evaluation metrics, for Spectral clustering (Spearman>0) for hESC at 1Mbp resolution. **(a)** Distribution of the score from different evaluation metrics (Davies-Bouldin index, Delta contact counts, and Silhouette index) for random cluster assignments (box plot) compared to actual scores (red star). **(b)** The distribution of positive Spearman correlation between regions within cluster C0 and C1 and between region pairs, where one member is from C0 and another member is from C1. **(c)** Same as **b** for all pairs of clusters.

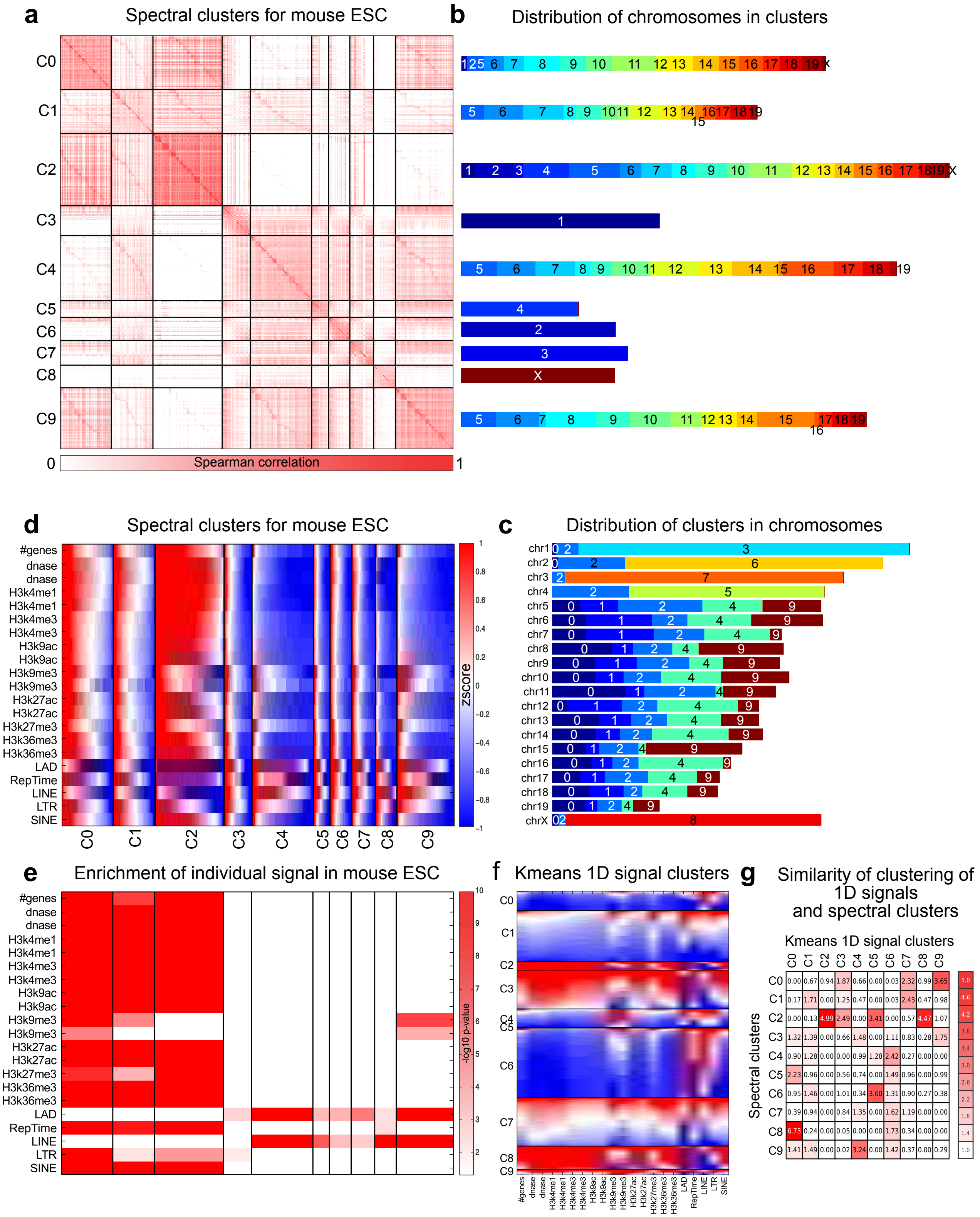
a
Spectral clusters for mouse ESC
Spearman correlation
b
Distribution of chromosomes in clusters
d
Spectral clusters for mouse ESC
zscore
c
Distribution of clusters in chromosomes
e
Enrichment of individual signal in mouse ESC
-log10 p-value
f
Kmeans 1D signal clusters
g
Similarity of clustering of 1D signals and spectral clusters
Kmeans 1D signal clusters
Spectral clusters
enrichment ratio

Figure S6: Spectral clustering results (Spearman>0) for mouse ESC Hi-C data. The figure follows the same legend as **Figure 2**. In panels **d** and **e**, repeated signals indicate that they come from 2 different ES cell lines.

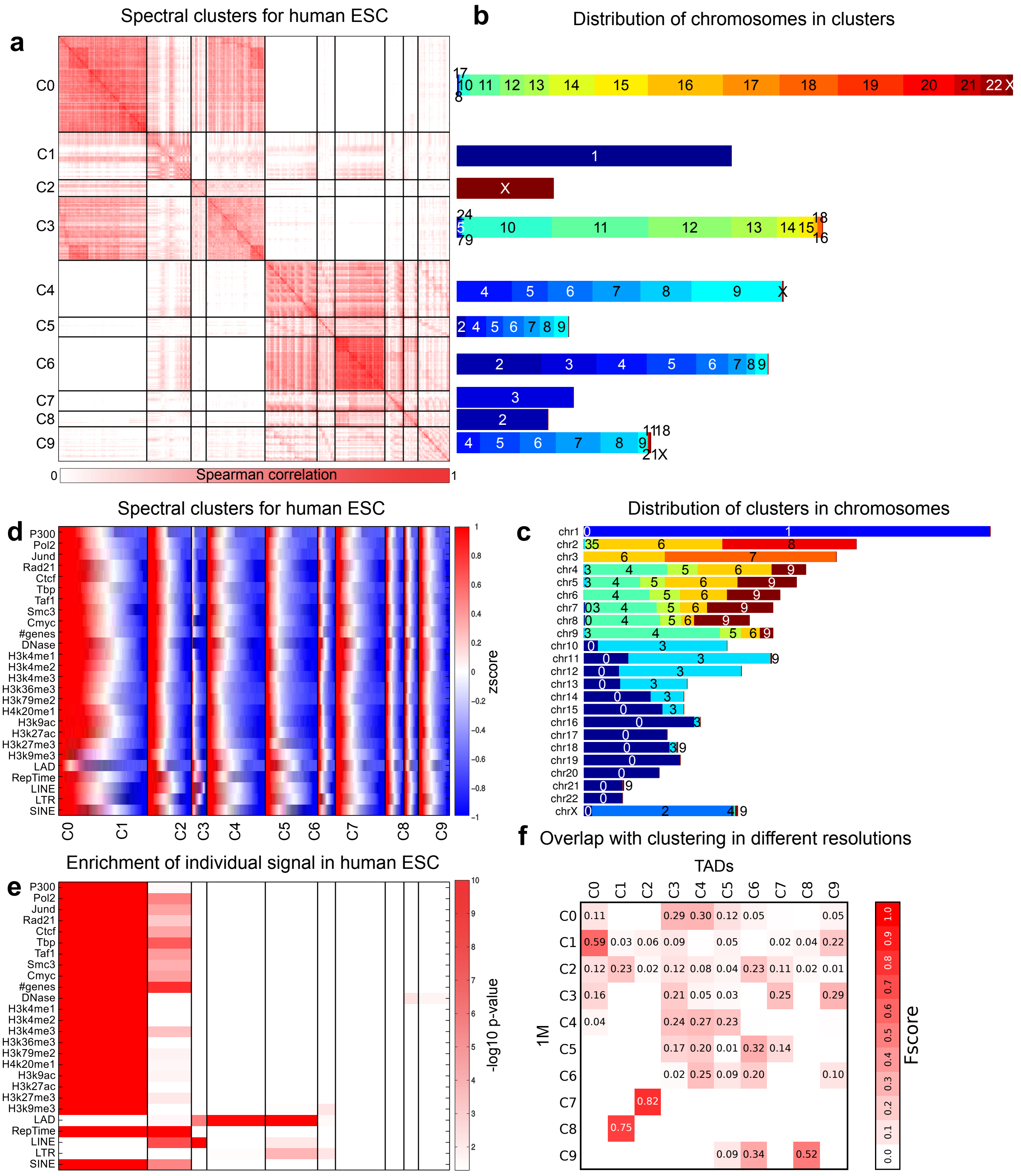
Spectral clusters for human ESC
a
b
Distribution of chromosomes in clusters
C0
C1
C2
C3
C4
C5
C6
C7
C8
C9
Spearman correlation
d
Spectral clusters for human ESC
zscore
c
Distribution of clusters in chromosomes
P300
Pol2
Jund
Rad21
Ctcf
Tbp
Taf1
Smc3
Cmyc
#genes
DNase
H3k4me1
H3k4me2
H3k4me3
H3k36me3
H3k79me2
H4k20me1
H3k9ac
H3k27ac
H3k27me3
H3k9me3
LAD
RepTime
LINE
LTR
SINE
e
Enrichment of individual signal in human ESC
-log10 p-value
f
Overlap with clustering in different resolutions
TADs
1M
Fscore

Figure S7: Spectral clustering of human ESC Hi-C data using TAD regions as bins. **(a)** The heatmap of Spearman correlation matrix of contact counts between TADs. **(b)** The bar plot shows the distribution of chromosomes in the clusters. **(c)** The bar plot shows the distribution of clusters in the chromosomes. **(d)** Different regulatory features grouped according to the spectral cluster assignments. **(e)** -log10 of KS-test $p$-values. **(f)** Overlap of the TAD clusters and fixed bin clusters at 1Mbp resolution. Each heatmap shows the F-score based overlap between each pair of clusters. The more red the intensity the greater the overlap.

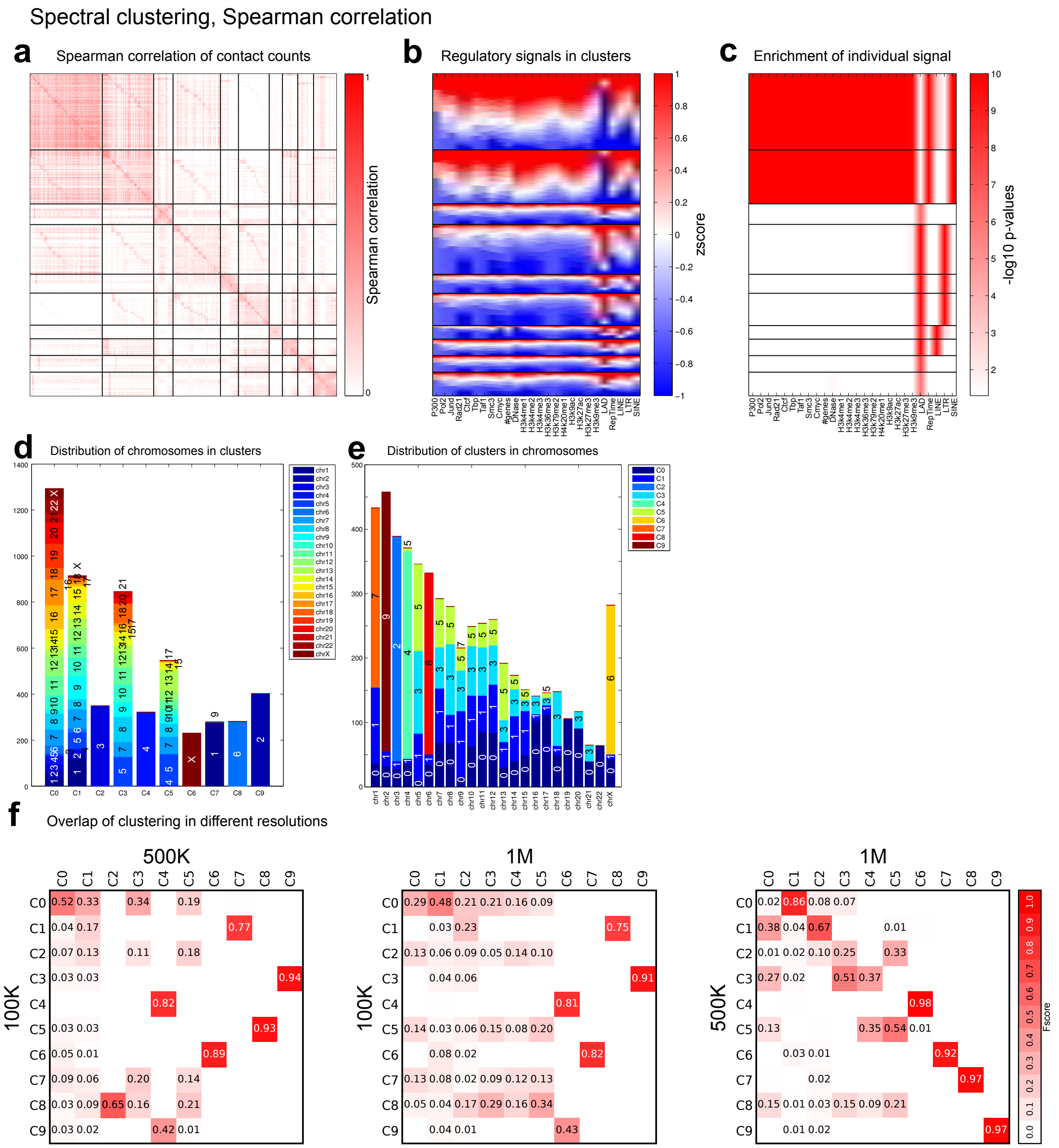
Spectral clustering, Spearman correlation
a
Spearman correlation of contact counts
Spearman correlation
b
Regulatory signals in clusters
zscore
c
Enrichment of individual signal
-log10 p-values
d
Distribution of chromosomes in clusters
e
Distribution of clusters in chromosomes
f
Overlap of clustering in different resolutions
500K
1M
1M
100K
100K
500K
Fscore

Figure S8: Spectral clustering results of human hESC data at different resolutions. **(a)** The heatmap of Spearman correlation matrix of contact counts at 500Kbp bins, reordered according to the cluster assignments. **(b)** The enrichment of different regulatory features reordered according to the cluster assignments. **(c)** -log10 of KS-test $p$-values of each feature inside a cluster versus outside. **(d)** The distribution of chromosomes in each cluster. **(e)** The distribution of clusters in each chromosome. **(f)** The overlap of clusters at different resolutions. Each heatmap shows the F-score based overlap between pairs of clusters at two different resolutions. The more red the intensity the greater the overlap.

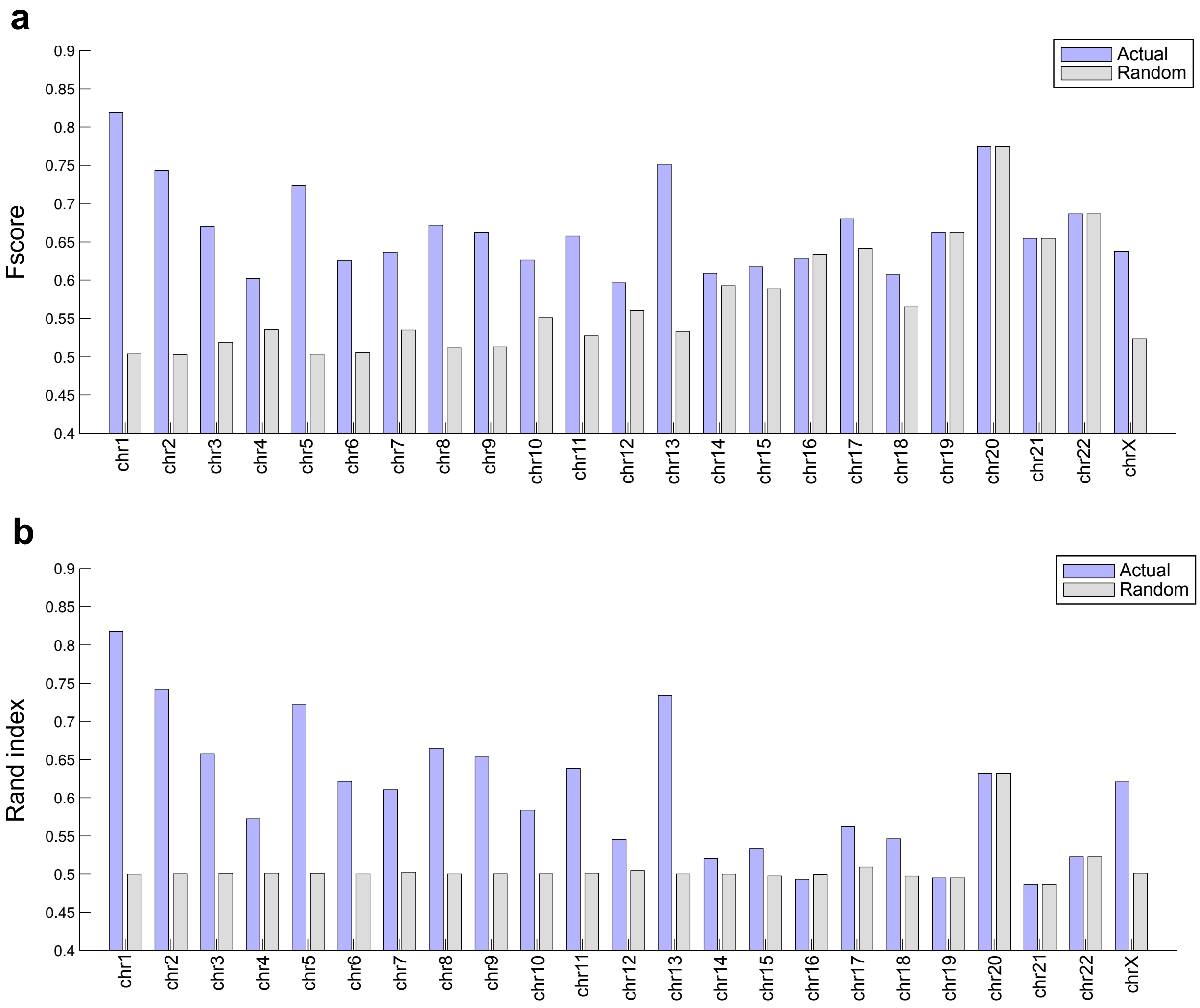
a
Fscore
0.9
0.85
0.8
0.75
0.7
0.65
0.6
0.55
0.5
0.45
0.4
Actual
Random
chr1
chr2
chr3
chr4
chr5
chr6
chr7
chr8
chr9
chr10
chr11
chr12
chr13
chr14
chr15
chr16
chr17
chr18
chr19
chr20
chr21
chr22
chrX
b
Rand index
0.9
0.85
0.8
0.75
0.7
0.65
0.6
0.55
0.5
0.45
0.4
Actual
Random
chr1
chr2
chr3
chr4
chr5
chr6
chr7
chr8
chr9
chr10
chr11
chr12
chr13
chr14
chr15
chr16
chr17
chr18
chr19
chr20
chr21
chr22
chrX

Figure S9: Similarity of chromosomal compartments and genome-wide spectral clusters in human ESC Hi-C data. **(a)** Shown is the F-score based overlap between the compartments and cluster assignments. **(b)** Shown are the Rand index based overlap between compartments and cluster assignments. The similarity of clusters and chromosomal compartments are greater than we expect by random for most chromosomes. The exceptions are chromosomes 16, and 19-22, where the whole chromosome was put in one cluster.

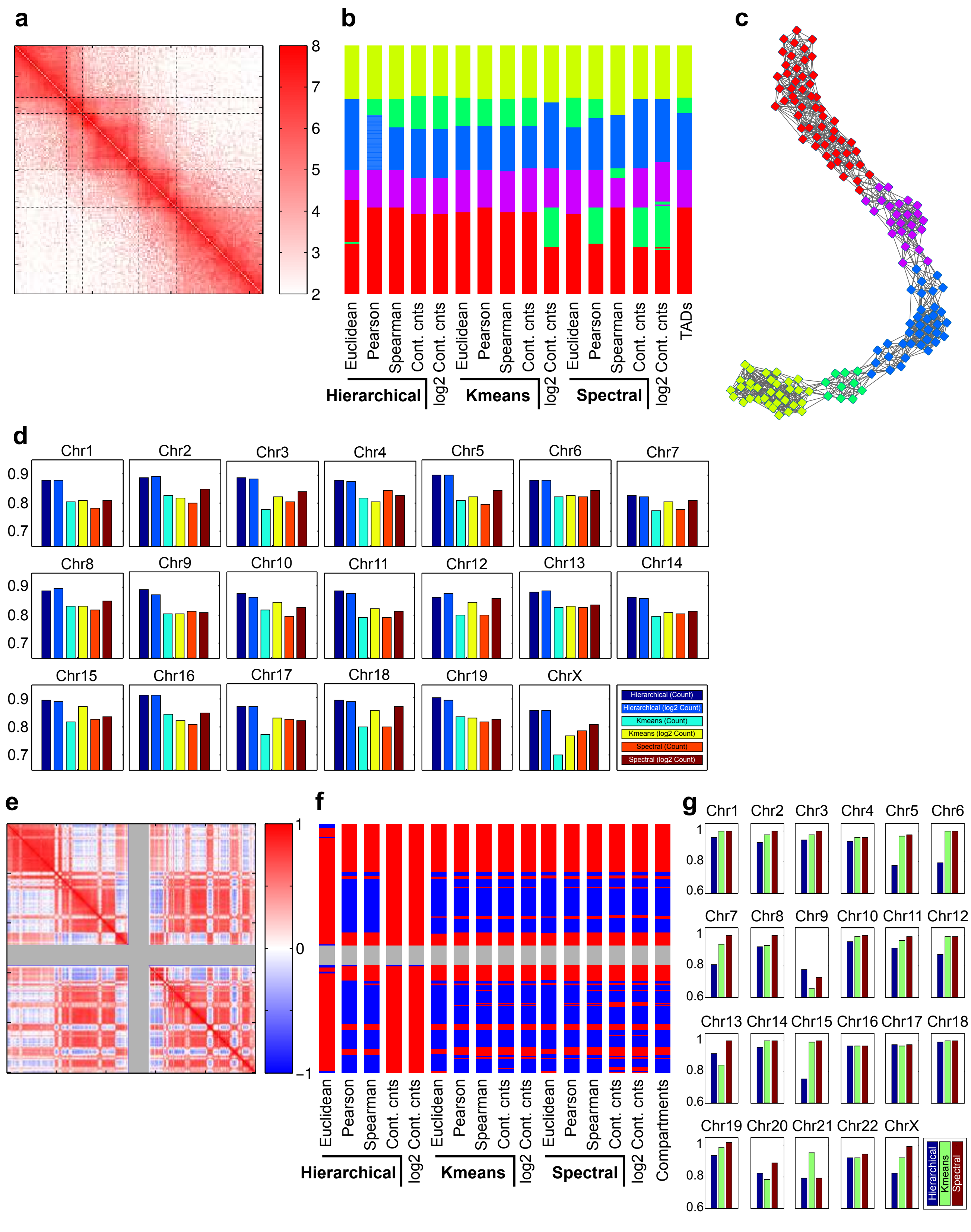
a
8
7
6
5
4
3
2
b
Euclidean
Pearson
Spearman
Cont. cnts
log2 Cont. cnts
Euclidean
Pearson
Spearman
Cont. cnts
log2 Cont. cnts
Euclidean
Pearson
Spearman
Cont. cnts
log2 Cont. cnts
TADs
Hierarchical
Kmeans
Spectral
c
d
Chr1 Chr2 Chr3 Chr4 Chr5 Chr6 Chr7
Chr8 Chr9 Chr10 Chr11 Chr12 Chr13 Chr14
Chr15 Chr16 Chr17 Chr18 Chr19 ChrX
0.9
0.8
0.7
Hierarchical (Count)
Hierarchical (log2 Count)
Kmeans (Count)
Kmeans (log2 Count)
Spectral (Count)
Spectral (log2 Count)
e
1
0
−1
f
Euclidean
Pearson
Spearman
Cont. cnts
log2 Cont. cnts
Euclidean
Pearson
Spearman
Cont. cnts
log2 Cont. cnts
Euclidean
Pearson
Spearman
Cont. cnts
log2 Cont. cnts
Compartments
Hierarchical
Kmeans
Spectral
g
Chr1 Chr2 Chr3 Chr4 Chr5 Chr6
Chr7 Chr8 Chr9 Chr10 Chr11 Chr12
Chr13 Chr14 Chr15 Chr16 Chr17 Chr18
Chr19 Chr20 Chr21 Chr22 ChrX
1
0.8
0.6
Hierarchical
Kmeans
Spectral

Figure S10: Testing the ability of different clustering methods to recover TADs and compartments. **(a)** Heatmap of log2 of contact counts spanning 3Mb-9.5Mb of mouse chromosome 1 at 40Kbp resolution (corresponding to first 5 TADs identified from mouse ESC Hi-C data). Dotted lines correspond to the boundaries of the TADs. **(b)** The clustering result of these regions from 3 different methods and 5 different distance metrics (the same methods reported in **Figure 1**) with $k = 5$. **(c)** The graph representation of the same regions. Edges correspond to top 10% of the contact counts and colors correspond to TAD assignments. **(d)** The percentage of overlap between mouse ESC TADs and cluster assignments from different clustering methods, displayed per chromosome. **(e)** Heatmap of Spearman correlation of normalized contact counts from human ESC Hi-C data for chromosome 1. Grey areas correspond to regions with no measured contact counts in chromosome 1. **(f)** The clustering results of chromosome 1 of human ESC at 1Mbp resolution from 3 different methods and 5 different distance metrics with $k = 2$. **(g)** Percentage overlap of compartments and cluster assignments from different clustering methods for all the human chromosomes.

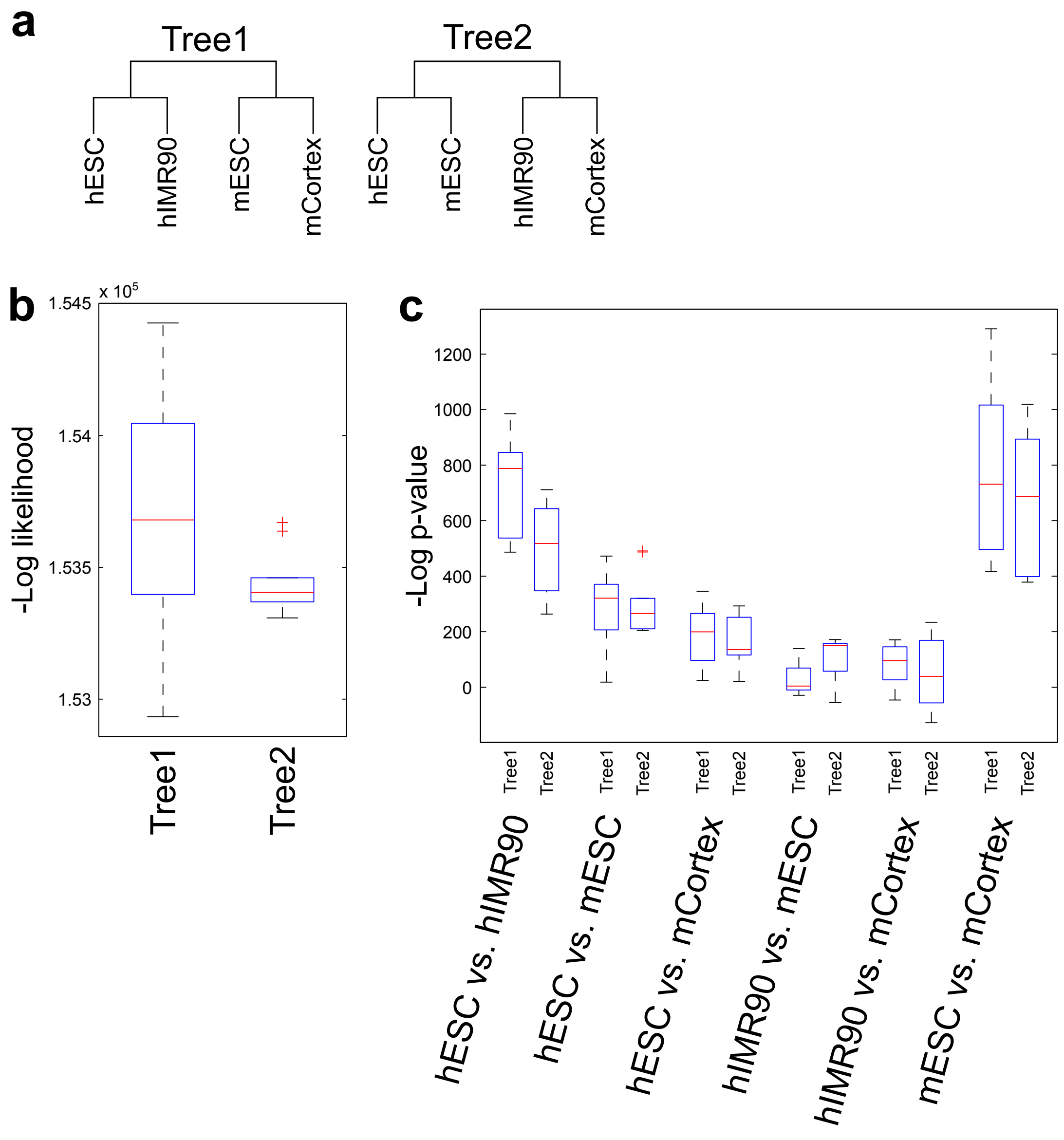
a
Tree1
Tree2
hESC
hIMR90
mESC
mCortex
hESC
mESC
hIMR90
mCortex
b
x 10^5
1.545
1.54
1.535
1.53
-Log likelihood
Tree1
Tree2
c
-Log p-value
1200
1000
800
600
400
200
0
Tree1
Tree2
hESC vs. hIMR90
hESC vs. mESC
hESC vs. mCortex
hIMR90 vs. mESC
hIMR90 vs. mCortex
mESC vs. mCortex

Figure S11: The effect of alternative phylogenetic tree structure on Arboretum-Hi-C. **(a)** Two alternative structures of relatedness between the Hi-C datasets examined in this work. **(b)** The reported -log likelihood of the models from 10 random initializations using each of the tree structures. **(c)** Conservation scores for pairs of cell lines (between and within species) using the two tree structures. Each box corresponds to the distribution of the conservation scores from 10 different runs of the Arboretum-Hi-C.

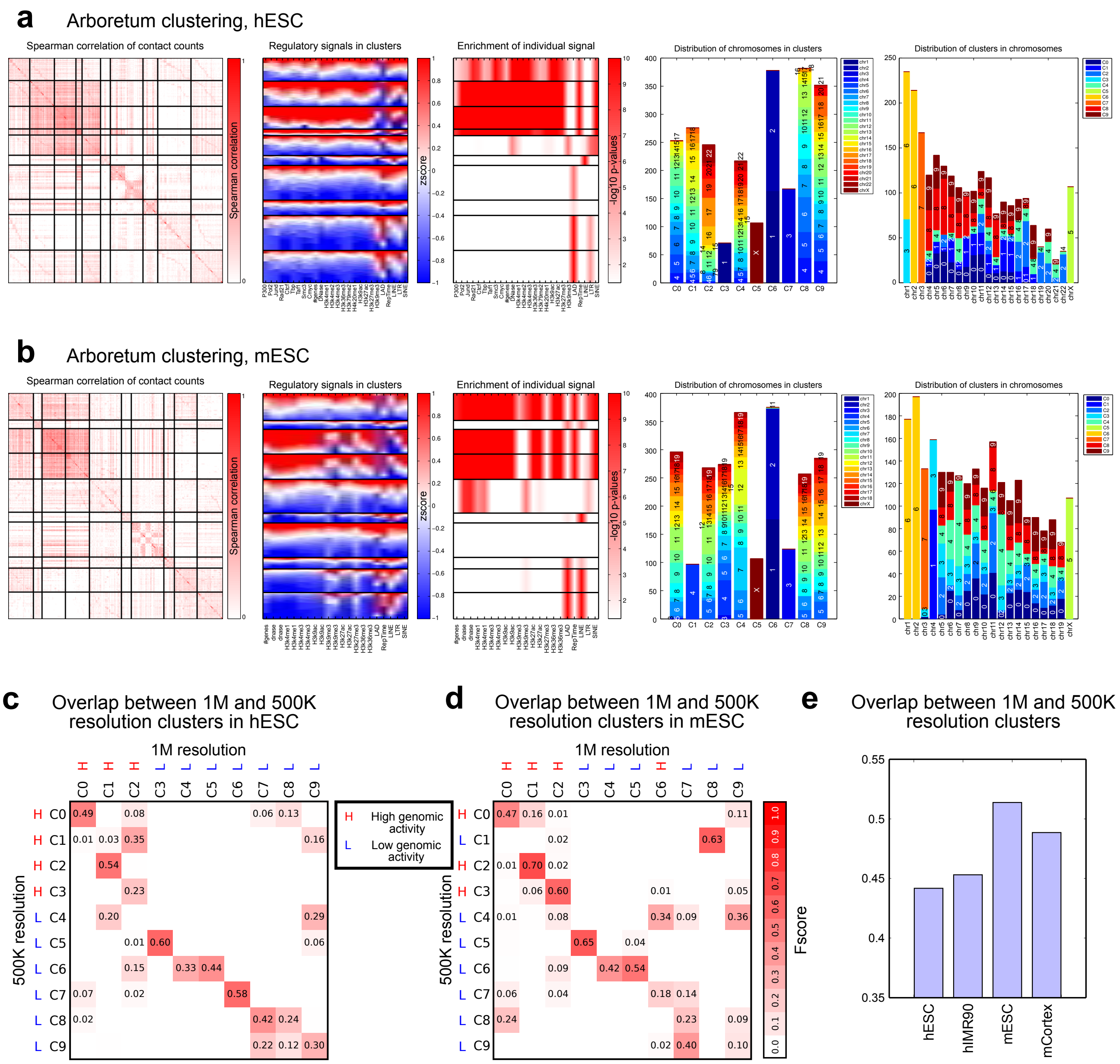
a
Arboretum clustering, hESC
Spearman correlation of contact counts
Spearman correlation
Regulatory signals in clusters
zscore
Enrichment of individual signal
-log10 p-values
Distribution of chromosomes in clusters
Distribution of clusters in chromosomes
b
Arboretum clustering, mESC
Spearman correlation of contact counts
Spearman correlation
Regulatory signals in clusters
zscore
Enrichment of individual signal
-log10 p-values
Distribution of chromosomes in clusters
Distribution of clusters in chromosomes
c
Overlap between 1M and 500K resolution clusters in hESC
1M resolution
500K resolution
H High genomic activity
L Low genomic activity
d
Overlap between 1M and 500K resolution clusters in mESC
1M resolution
500K resolution
Fscore
e
Overlap between 1M and 500K resolution clusters
hESC
hIMR90
mESC
mCortex

Figure S12: Arboretum-Hi-C clusters at 500Kbp resolution. **(a)** The first plot is the heatmap of Spearman correlation matrix of contact counts for orthologous regions in human ESCs, reordered according to the Arboretum-Hi-C cluster assignments. The second plot is the enrichment of different regulatory features reordered according to the cluster assignments. The third plot is -log10 of KS-test $p$-values to assess enrichment of signals in each cluster. The fourth plot is the distribution of chromosomes in each cluster, and the last plot is the distribution of clusters in each chromosome. **(b)** Same as **a** for mouse ESC Hi-C data. **(c)** Pairwise similarity of Arboretum-Hi-C clusters in human ESC at 500Kbp and 1Mbp resolutions. Values in the heatmap correspond to Fscore similarity of each pair of clusters. H and L correspond to high and low genomic activity in the cluster. **(d)** Same as **c** for mouse ESC. **(e)** Shown is the overlap (bar height) of Arboretum-Hi-C clusters at 500Kbp and 1Mbp resolutions for each of the four cell lines.

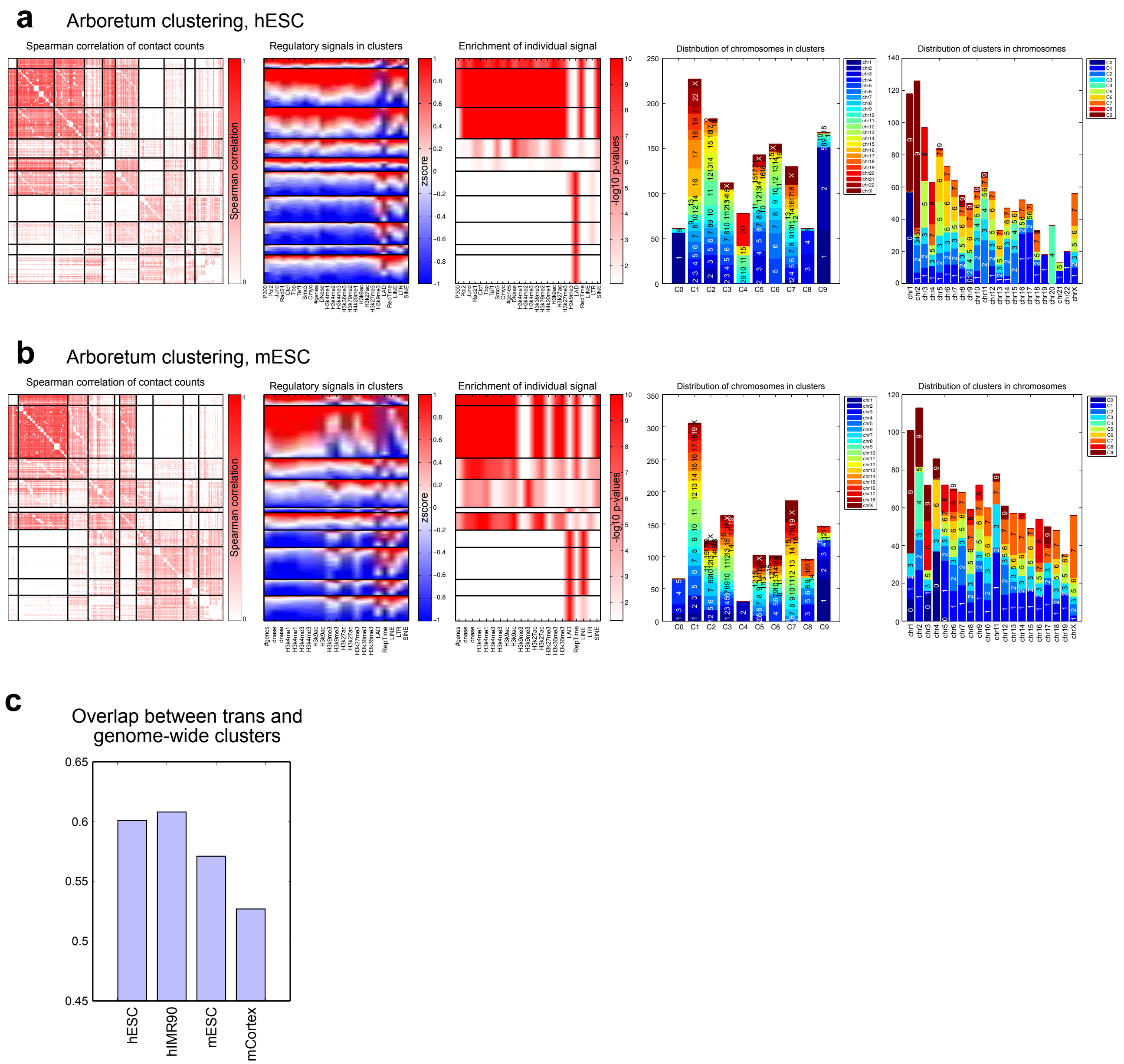
a
Arboretum clustering, hESC
Spearman correlation of contact counts
Spearman correlation
Regulatory signals in clusters
zscore
Enrichment of individual signal
-log10 p-values
Distribution of chromosomes in clusters
Distribution of clusters in chromosomes
b
Arboretum clustering, mESC
Spearman correlation of contact counts
Spearman correlation
Regulatory signals in clusters
zscore
Enrichment of individual signal
-log10 p-values
Distribution of chromosomes in clusters
Distribution of clusters in chromosomes
c
Overlap between trans and genome-wide clusters
hESC
hIMR90
mESC
mCortex

Figure S13: Enrichment of Arboretum-Hi-C clusters at 1Mbp resolution when clustering only inter-chromosomal (*trans*) interaction, and their overlap with Arboretum-Hi-C clusters. **(a)** The first plot is the heatmap of Spearman correlation matrix of contact counts for orthologous regions in human ESC Hi-C data, reordered according to the Arboretum-Hi-C cluster assignments. The second plot is the enrichment of different regulatory features reordered according to the cluster assignments. The third plot is -log10 of KS-test $p$-values. The fourth plot is the distribution of chromosomes in each cluster, and the last plot is the distribution of clusters in each chromosome. **(b)** The same as **a**, for mouse ESC Hi-C data. **(c)** The overlap of Arboretum-Hi-C clusters obtained using inter-chromosomal interactions and using both inter and intra-chromosomal interactions, for each of the four cell lines.

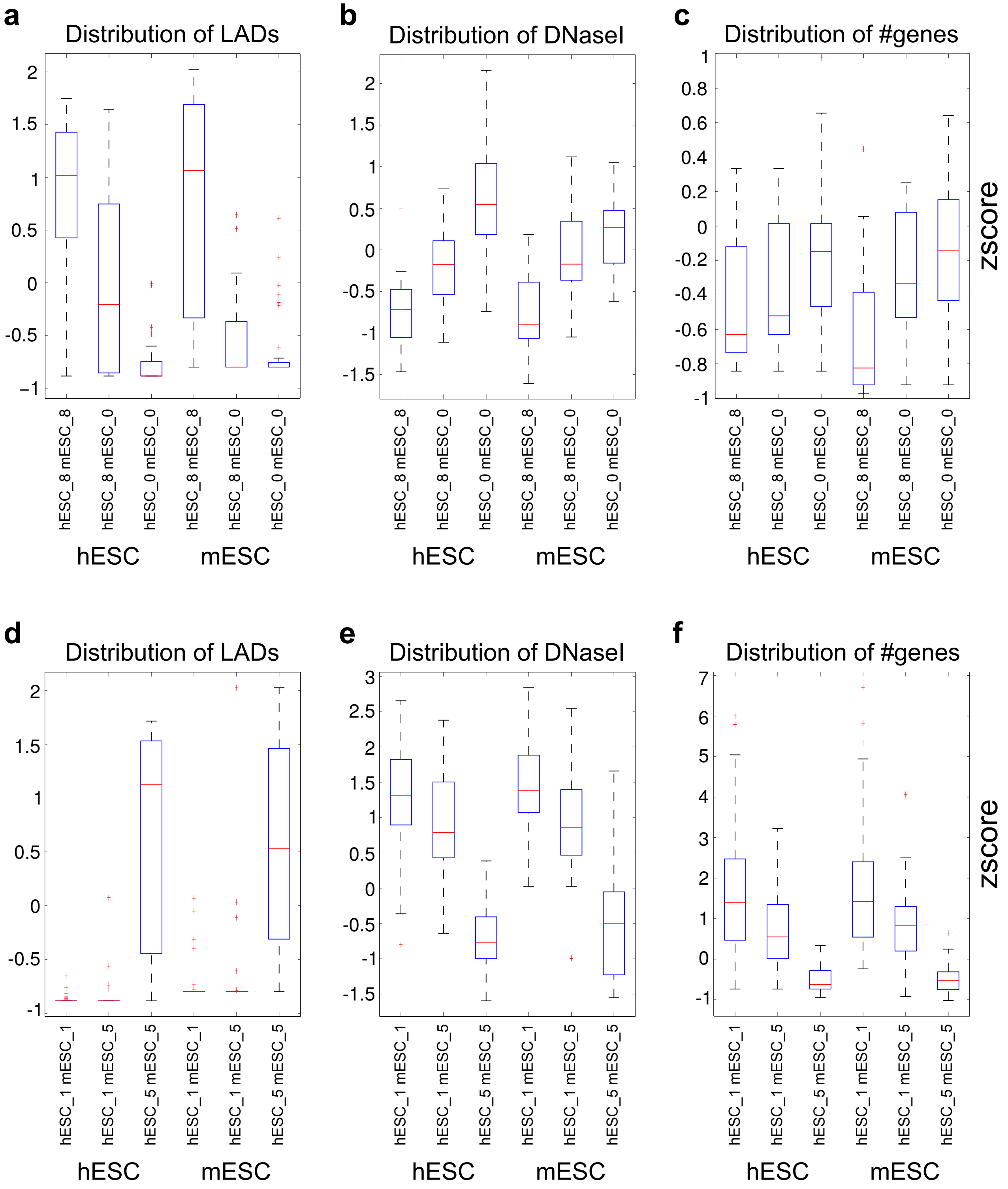
a
Distribution of LADs
b
Distribution of DNaseI
c
Distribution of #genes
zscore
hESC_8 mESC_8
hESC_8 mESC_0
hESC_0 mESC_0
hESC
mESC
d
Distribution of LADs
e
Distribution of DNaseI
f
Distribution of #genes
zscore
hESC_1 mESC_1
hESC_1 mESC_5
hESC_5 mESC_5
hESC
mESC

Figure S14: Distribution different regulatory signals in diverged and conserved regions between human and mouse ESC. **(a)** Distribution of LADs in regions that stay in cluster 8 (*hESC_8_mESC_8*), that stay in cluster 0 (*hESC_0_mESC_0*), and those that move from cluster 8 to 0 (*hESC_8_mESC_0*) in human and mouse ESC. **(b)** Distribution of DNase I in the same regions as **a**. **(c)** Distribution of number of genes in the same regions as **a**. **(d)** Distribution of LADs in regions that stay in cluster 1 (*hESC_1_mESC_1*), stay in cluster 5 (*hESC_5_mESC_5*), and move from cluster 1 to 5 (*hESC_1_mESC_5*) in human and mouse ESC. **(e)** Distribution of DNase I in the same regions as **d**. **(f)** Distribution of number of genes in the same regions as **d**.

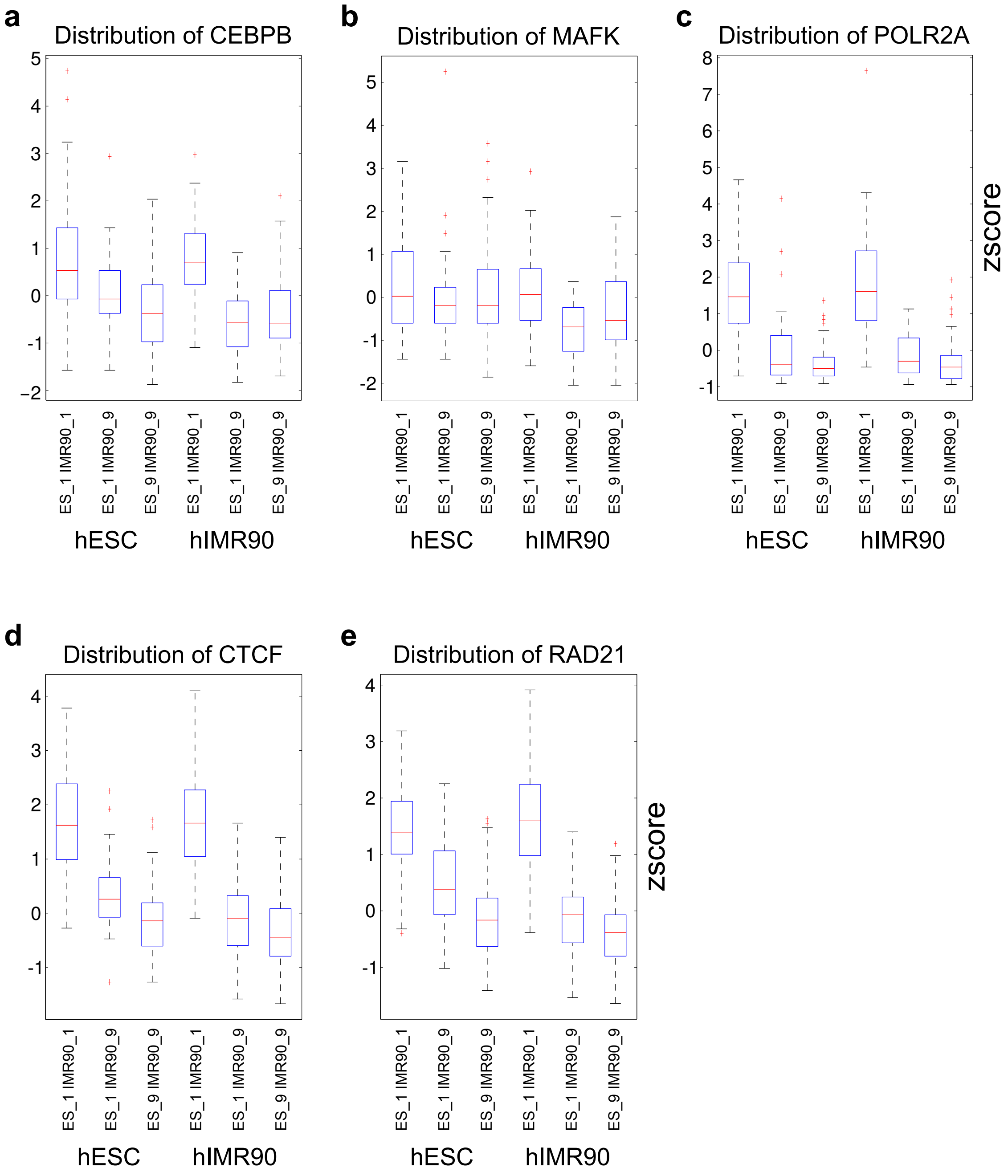
a
Distribution of CEBPB
b
Distribution of MAFK
c
Distribution of POLR2A
zscore
d
Distribution of CTCF
e
Distribution of RAD21
zscore
ES_1 IMR90_1
ES_1 IMR90_9
ES_9 IMR90_9
hESC
hIMR90

Figure S15: Distribution of different TF ChIP-seq peak calls in regions that stay or change their cluster/module assignment between cell lines. **(a)** CEBPB, **(b)** MAFK, **(c)** POLR2A, **(d)** CTCF, **(e)** RAD21. Each panel shows the differential enrichment of these signals in regions of clusters 1 and 9 in human ESC and IMR90 cell lines (Similar legend **Figure 6**). There is a significant change in the distribution number of peaks between diverged regions and regions of clusters 1 and 9 for CTCF, RAD21 and CEBPB while the difference in distribution for MAFK and POLR2A is not as significant.

# 3 Supplementary Tables

| Comparison Type | Overlap | Random Overlap |
|---|---|---|
| 1Mbp vs TAD | 43% | 21%±1 |
| 500Kbp vs 100Kbp | 64% | 33%±0.5 |
| 1Mbp vs 100Kbp | 53% | 32%±0.5 |
| 1Mbp vs 500Kbp | 71% | 23%±1 |
| 1Mbp *cis-trans* vs 1Mbp *trans* | 59% | 24%±0.5 |

Table S1: Estimated overlap between clusterings obtained at different resolutions and bin definitions

# 4 Supplementary Dataset

**Supplementary Dataset 1** includes **Additional Files 1** and **2**. These files include the cluster assignments of different methods on a single cell line, the cluster assignments of Arboretum-Hi-C on all cell lines, and feature vectors for human and mouse ESC.

# References


[1] J. R. Dixon, S. Selvaraj, F. Yue, A. Kim, Y. Li, Y. Shen, M. Hu, J. S. Liu, and B. Ren. Topological domains in mammalian genomes identified by analysis of chromatin interactions. *Nature*, 485(7398):376–380, May 2012.

[2] E. Lieberman-Aiden, N. L. van Berkum, L. Williams, M. Imakaev, T. Ragoczy, A. Telling, I. Amit, B. R. Lajoie, P. J. Sabo, M. O. Dorschner, R. Sandstrom, B. Bernstein, M. A. Bender, M. Groudine, A. Gnirke, J. Stamatoyannopoulos, L. A. Mirny, E. S. Lander, and J. Dekker. Comprehensive Mapping of Long-Range Interactions Reveals Folding Principles of the Human Genome. *Science*, 326(5950):289–293, Oct. 2009.
